# Prognostic discrimination of SOFA-2 vs. SOFA-1 and incremental information from cardiovascular scoring and vasoactive support intensity in patients with sepsis receiving early vasoactive therapy

**DOI:** 10.3389/fcvm.2026.1918792

**Published:** 2026-09-30

**Authors:** Tianci Huang, Mingyuan Pan, Lifan Pan, Peng Liang, Chenglin Mou, Junyu Lu

**Affiliations:** 1 Department of Critical Care Medicine, The Second Affiliated Hospital of Guangxi Medical University, Nanning, Guangxi, China; 2 Department of Emergency Medicine, Wuming Hospital of Guangxi Medical University, Nanning, Guangxi, China; 3 Department of Critical Care Medicine, Wuming Hospital of Guangxi Medical University, Nanning, Guangxi, China

**Keywords:** intensive care, norepinephrine-equivalent burden, sepsis, septic shock, SOFA-2, vasoactive drugs

## Abstract

**Background:**

Patients with sepsis who require vasoactive support during the first 24 h of intensive care represent a heterogeneous population. It is uncertain whether the Sequential Organ Failure Assessment (SOFA)-2 score improves mortality discrimination compared with the SOFA-1 score and whether observed circulatory support intensity provides complementary prognostic information.

**Methods:**

This multicohort observational study used Medical Information Mart for Intensive Care IV (MIMIC-IV) for a 24-h conditional landmark analysis. Patients alive and still in the ICU beyond 24 h were followed from ICU admission +24 h through ICU day 28 for reconstructed all-cause mortality. We compared SOFA-1 and SOFA-2, evaluated SOFA-2 total and cardiovascular scores, analyzed first-24-h five-agent norepinephrine-equivalent peak burden (norepinephrine, epinephrine, dopamine, phenylephrine, and vasopressin), and internally evaluated an exploratory drug-composition model. Fixed strata were assessed directionally in eICU Collaborative Research Database and an independent-center high-risk stress test cohort with cohort-specific endpoints.

**Results:**

The MIMIC-IV 24-h landmark cohort included 11,892 patients and 2,687 post-landmark deaths. SOFA-2 showed no observed discrimination advantage over SOFA-1 (area under the receiver operating characteristic curve, AUROC, 0.731 vs. 0.732; difference, −0.0005; 95% confidence interval, −0.0086 to 0.0076; *P* = 0.908). The SOFA-2 cardiovascular score had lower discrimination than the SOFA-1 cardiovascular score (0.663 vs. 0.676; *P* = 0.006). Mortality was 51.94% for the exploratory SOFA-2 ≥12 group and 17.09% below 12, and increased from 11.30% to 47.45% across four study-defined peak burden categories. The SOFA-2-plus-drug-composition model had an apparent AUROC of 0.768 and a bootstrap-corrected AUROC of 0.767, but it was not externally validated. Fixed strata preserved the direction of risk ranking in both external cohorts, whereas absolute risk and calibration differed.

**Conclusion:**

SOFA-2 did not show an observed overall mortality discrimination advantage over SOFA-1 in this population. SOFA-2 total and cardiovascular scores, together with observed vasoactive support intensity, provided complementary prognostic description at 24 h. The ≥12 rule is exploratory, and drug-related associations do not establish causal treatment effects or clinical decision utility.

## 1 Background

Sepsis remains a major cause of death in the intensive care unit (ICU). Early recognition of high-risk patients can inform clinical assessment, treatment monitoring, and stratification in clinical research (1, 2). Among patients with sepsis who receive vasoactive therapy soon after ICU admission, mortality risk is related not only to overall organ dysfunction but also to the intensity of circulatory support, drug dose, and the complexity of combination therapy. A binary indicator of vasoactive drug use may therefore obscure clinically meaningful differences in risk (3, 4).

The Sequential Organ Failure Assessment (SOFA) score is widely used to describe organ dysfunction, identify sepsis, and stratify risk. SOFA-2 updated the scoring rules for its six organ domains to reflect contemporary ICU practice (5, 6).

Whether SOFA-2 improves overall mortality discrimination compared with SOFA-1 remains uncertain in the selected population that receives vasoactive therapy during the first 24 h and survives to the 24-h assessment point. Total-score area under the receiver operating characteristic curve (AUROC) alone also cannot determine whether the updated cardiovascular score or directly observed support intensity adds clinically interpretable prognostic information (7–9).

The SOFA-2 cardiovascular score discretizes mean arterial pressure (MAP), vasoactive or inotropic support, and mechanical circulatory support. In contrast, five-agent norepinephrine-equivalent (NE-equivalent) peak burden retains continuous dose resolution for combined drug support, while individual drug use indicators describe treatment composition. These measures overlap but are not identical, and their associations must be separated from causal effects of treatment selection (10–12).

We hypothesized that, among patients with sepsis who received vasoactive therapy during the first 24 h and were alive and still in the ICU beyond 24 h, SOFA-2 would not necessarily improve overall mortality discrimination compared with SOFA-1, whereas the SOFA-2 cardiovascular score and observed vasoactive support intensity would provide complementary prognostic information. We tested this hypothesis in a Medical Information Mart for Intensive Care IV (MIMIC-IV) 24-h landmark cohort, internally evaluated an exploratory drug-composition model, and assessed directional transportability of fixed strata in eICU Collaborative Research Database (eICU) and an independent-center high-risk stress test cohort.

## 2 Methods

### 2.1 Study design and data sources

This multicohort observational study used MIMIC-IV for the primary 24-h landmark analysis and internal validation of prediction models. The eICU database was used to assess transportability of the fixed SOFA stratification rule with a database-specific endpoint, and an anonymized independent center was used to stress test the rule in a high-risk case mix. Cohort-specific operational definitions, screening roles, and endpoints are summarized in Supplementary Table S1.

MIMIC-IV v3.1 (PhysioNet; admissions spanning 2008–2022; released 11 October 2024; dataset DOI: 10.13026/kpb9-mt58) (13) and eICU-CRD v2.0 (United States ICU admissions from 2014 to 2015) (14) are deidentified critical care databases available through PhysioNet to credentialed users who complete the required training and sign the applicable data use agreements. Cases in MIMIC-IV were identified from the official mimiciv_derived.sepsis3 derived table and retained locked outputs. Johnson et al. (15) describe the general methodology of the mimic code repository, but the retained materials do not record the exact commit used to generate the derived table; therefore, no unverifiable claim about the source code version is made. Investigators obtained database access after completing the required training and certification (certificate no. 67457488; certified 25 January 2025). The anonymized independent-center cohort included eligible ICU patients admitted from 1 January 2023 through 1 April 2026. Twenty-eight-day all-cause mortality was determined for every included patient. The relevant institutional ethics committee approved the study [approval no. 2024-KY(0912)]. Reporting followed the strengthening the reporting of observational studies in epidemiology (STROBE) statement (16), and the transparent reporting of a multivariable prediction model for individual prognosis or diagnosis (TRIPOD) checklist was completed.

### 2.2 Study population

Each cohort used a case identification method suited to its data structure.

MIMIC-IV used the standardized mimiciv_derived.sepsis3 cohort (15, 17). When antibiotics preceded culture sampling, a culture was required within 24 h after antibiotic initiation; when culture sampling preceded antibiotics, antibiotics were required within 72 h after culture collection. The SOFA assessment window extended from 48 h before to 24 h after suspected infection. The primary cohort imposed no additional restriction on the timing of suspected infection relative to ICU admission.

Because eICU could not reproduce the antibiotic–culture timing pair in an isomorphic manner, cases were identified using a structured diagnosis of sepsis or septic shock within 24 h before or after ICU admission, together with a calculable first-day SOFA score of ≥2. This was a database-specific case identification strategy rather than a fully isomorphic implementation of the Sepsis-3 derived algorithm.

At the independent center, clinicians reviewed individual cases for confirmed or suspected infection accompanied by an increase of ≥2 SOFA points from baseline and made the final determination. Of 615 clinically reviewed records, patients with complete locked SOFA-2 source fields and evidence of vasoactive drug exposure during the first 24 h were eligible. Complete SOFA-2 source fields were available for 492 patients, all of whom met the exposure criterion; the final cohort therefore comprised 492 patients. Admissions occurred from 1 January 2023 through 1 April 2026, and 28-day all-cause mortality was ascertainable for all included patients. This cohort retained locked scoring and early exposure definitions and was used only for a directional stress testing, not for direct comparison of absolute risk with MIMIC-IV.

Baseline eligibility criteria were age ≥18 years; an ICU stay extending beyond 24 h; survival beyond 24 h after ICU admission; the first eligible ICU admission; receipt of at least one target vasoactive drug during hours 0–24; and ascertainable SOFA-1, SOFA-2, and cohort-specific outcomes. In MIMIC-IV, survival beyond the 24-h landmark was verified directly against death timestamps; 105 deaths at or before the landmark were excluded. The eICU and independent-center cohorts likewise comprised patients alive and still in the ICU beyond 24 h, permitting fixed-stratum assessment at the same 24-h index, although their outcome definitions remained cohort-specific.

No formal sample size calculation was performed. Each cohort included all patients who met the relevant eligibility criteria during the study period and had the required study variables available.

### 2.3 Data processing and missing data

Before analysis, variable provenance was checked in each cohort, measurement units were harmonized, and physiologic plausibility was assessed. Patient identifiers, outcomes, SOFA scores, and NE-equivalent exposures were not imputed. Assessable first-day Glasgow Coma Scale (GCS) was missing for 29.99% of the MIMIC-IV cohort; derived GCS was therefore not presented as observed GCS. Variable- and patient-level missingness in the landmark cohort is shown in Supplementary Figure S1, and variable completeness in the source cohort is reported in Supplementary Table S3. Mechanical ventilation in the main table was strictly defined as invasive ventilation or tracheostomy during the first 24 h.

In the primary MIMIC-IV landmark adjustment models, only lactate was multiply imputed using chained equations. A total of 20 imputed datasets were generated and combined using Rubin's rules. A complete-case analysis was performed as a sensitivity analysis (Supplementary Table S20).

### 2.4 Infection source

Infection sources were extracted from available structured diagnoses or clinical adjudication records and could be recorded as multiple non-mutually exclusive categories. The independent center classified pulmonary, urinary tract, intra-abdominal or hepatobiliary, central nervous system, and other or unknown infection, but did not separately collect bloodstream/endovascular or skin/soft-tissue/bone/joint infections. Categories that were not separately collected were not treated as absence of infection or as 0 cases. Blood culture pathogen indicators were not used to infer a primary bloodstream infection.

### 2.5 SOFA scoring and risk stratification

SOFA-1 was calculated according to the original definition (18), while the locked SOFA-2 scoring framework was aligned with the JAMA 2025 definition (5, 6). MIMIC-IV retained executable structured query language (SQL) code and quality-control outputs for all six domains, whereas the independent center retained only partial source fields and the current eICU cohort retained locked score fields. We therefore verified arithmetic agreement and evaluated the locked scores prognostically but do not claim fully equivalent event-level implementation across cohorts; organ domain rules and reproducibility status are detailed in Supplementary Table S9.

The 0–4 rules for the SOFA-2 cardiovascular score are provided in Supplementary Table S16. Only eligible vasopressors or inotropes administered by continuous intravenous infusion for at least 1 h contributed to the score. Dobutamine (item id 221653) and milrinone (item id 221986) were classified as other vasopressors/inotropes but were excluded from the five-agent NE-equivalent formula. Mechanical support comprised a venoarterial extracorporeal membrane oxygenation system, an intra-aortic balloon pump, a left ventricular assist device, and a microaxial flow pump. The dopamine-only thresholds for scores of 2, 3, and 4 were ≤20, >20–40, and >40 μg/kg/min, respectively.

Integer SOFA-2 cutpoints were scanned using the maximum log-rank statistic in the MIMIC-IV 24-h landmark cohort. The maximum statistic occurred at a SOFA-2 total score of ≥12, which was therefore designated the exploratory high-risk rule. The distribution of each integer SOFA-2 score and its corresponding post-landmark 28-day all-cause mortality are reported in Supplementary Table S11. When the maximum log-rank cutpoint-selection procedure was repeated in 1,000 bootstrap resamples, the two most frequently selected SOFA-2 cutpoints were ≥12 (65.4% of resamples) and ≥11 (28.7%). This rule was not prespecified and should not be considered universal or therapeutic.

The fixed ≥12 rule was applied to the eICU and independent-center cohorts at the 24-h assessment point without reestimation. Both external cohorts required survival and continued ICU stay beyond 24 h. The adjacent cutpoint of ≥11 was retained only as a sensitivity analysis. SOFA-2 cardiovascular scores were grouped as 0–2 and 3–4. External analyses assessed only the direction of risk ranking and descriptive calibration under cohort-specific endpoints; neither screening criteria nor rule parameters were reestimated.

### 2.6 Vasoactive drug exposure

In MIMIC-IV, the first-24-h five-agent NE-equivalent peak burden was recalculated from original administration records for norepinephrine, epinephrine, dopamine, phenylephrine, and vasopressin. The formula was as follows: NE-equivalent dose = norepinephrine + epinephrine + dopamine/100 + phenylephrine/10 + vasopressin (U/min) × 2.5 (19, 20). All doses except vasopressin were standardized to μg/kg/min. Vasopressin recorded in U/h was divided by 60 to obtain U/min. For example, 2.4 U/h equals 0.04 U/min and corresponds to an NE-equivalent dose of 0.10 μg/kg/min.

The primary exposure was constructed from positive-rate continuous-infusion intervals in the MIMIC-IV-derived vasoactive drug table within 0–24 h after ICU admission. When several drugs were administered concurrently, their converted doses were summed, and the maximum five-agent combined burden during the first 24 h was retained as the peak intensity of circulatory support. The primary definition allowed brief or fragmented positive-rate intervals to contribute to the maximum-burden calculation. True bolus administrations could not be identified reliably as separate exposures in the available records and were not classified separately. This measure represents the highest observed combined support intensity during the first 24 h; it is not an average dose and does not capture exposure duration or trajectories of escalation and deescalation.

To describe the gradient between vasoactive support intensity and mortality, the first-24-h five-agent NE-equivalent peak burden was summarized in four study-defined dose categories: ≤0.1, >0.1–0.3, >0.3–0.5, and >0.5 μg/kg/min. These categories were used for descriptive presentation and categorical association analyses and were not interpreted as optimal, treatment, or universal clinical thresholds. Incremental prediction analyses used continuous first-24-h five-agent NE-equivalent peak burden and therefore did not depend on these category boundaries. Vasopressin conversion was omitted in the catecholamine-only sensitivity analysis.

For the duration-qualified sensitivity analysis, intervals for the same drug separated by ≤5 min were merged into a single infusion episode. Only merged episodes lasting ≥60 min were retained to redefine the cohort and recalculate the first-24-h five-agent NE-equivalent peak burden (Supplementary Table S22). The complete first-24-h five-agent NE-equivalent peak burden could be reconstructed reliably only in MIMIC-IV; the external cohorts were used only to assess the fixed SOFA strata.

### 2.7 Outcomes

The primary MIMIC-IV outcome was all-cause mortality within 28 days after ICU admission. Exact admissions.deathtime was used when available; otherwise, patients.dod was used. An event was recorded when the interval from ICU in-time to death was 0–28 days. Discharge alive from the ICU or hospital did not result in early censoring. This implementation identified 2,792 deaths among 11,997 patients. eICU used a database-specific ICU 28-day endpoint derived from ICU discharge status and ICU length of stay; the independent center used traceable 28-day all-cause mortality. Cohort-specific endpoint definitions and inferential roles are summarized in Supplementary Table S8.

### 2.8 Landmark time, time zero, and risk set definition

The primary analysis used a 24-h conditional landmark design. Only patients who survived strictly beyond 24 h after ICU admission were included. Time zero was ICU admission plus 24 h, and follow-up continued through ICU day 28 (a maximum of 27 days after the landmark). All risk characterization variables were measured during 0–24 h after ICU admission. The study therefore characterizes risk at 24 h rather than predicting outcome immediately upon ICU admission.

### 2.9 Statistical analysis

Continuous variables are presented as mean ± standard deviation or median [interquartile range], as appropriate, and categorical variables as counts and percentages. Discrimination was evaluated using AUROC and 95% confidence interval (CI). Paired DeLong methods estimated both the SOFA-2-minus-SOFA-1 AUROC difference with its 95% CI and the two-sided *P*-value (21). The AUROC describes ranking, the Brier score quantifies overall prediction error, calibration assesses the agreement between predicted and observed risk, decision curve analysis (DCA) estimates potential net benefit under a stated threshold probability scenario, and ORs or risk differences summarize observational prognostic associations. These quantities were not treated as interchangeable.

In MIMIC-IV, we separately evaluated the SOFA-2 total score, SOFA-2 cardiovascular score, five-agent NE-equivalent peak burden, and drug composition. Prognostic association and predictive performance were treated as separate analytical aims. ORs, standardized risks, and risk differences described observational prognostic associations, whereas AUROC, Brier score, calibration, and decision curve analysis described discrimination or predictive performance (22, 23). None of these analyses estimated treatment effects. The composition model included only the SOFA-2 total score and five binary indicators for norepinephrine, epinephrine, dopamine, phenylephrine, and vasopressin use. The number of drug classes and the indicator for multiple drug use were excluded because they were deterministically redundant with the five individual indicators.

This model was developed and internally validated only in MIMIC-IV and was not refitted in the external cohorts.

Predictors for the composition model were selected *a priori* according to the research question and data availability, without univariable screening.

To assess the incremental value of cardiovascular support information beyond the non-cardiovascular SOFA-2 domains, M0 comprised the sum of the five non-cardiovascular SOFA-2 domains. We then added the cardiovascular score (M1), continuous five-agent NE-equivalent peak burden (M2), both variables (M3), or the five drug use indicators (M4). The complete SOFA-2 total score was M5. Models containing continuous first-24-h five-agent NE-equivalent peak burden used 11,890 patients and 2,686 events. The complete SOFA-2 total-score model and the drug-composition model underwent internal optimism correction with 1,000 bootstrap resamples. Model formulas, candidate denominators, and analysis denominators are reported in Supplementary Table S12.

Logistic regression was used to estimate ORs and 95% CIs. The exploratory total-score rule model was adjusted for age, sex, Charlson comorbidity index, and lactate. Cardiovascular-score and NE burden models additionally adjusted for non-cardiovascular SOFA-2, avoiding part–whole adjustment by reintroducing the exposure component through the total score. Only lactate was multiply imputed in 20 datasets, with estimates combined using Rubin's rules. In addition to adjusted ORs, we report marginal standardized mortality probabilities, adjusted risk differences, and 95% CIs. Sensitivity, specificity, predictive values, likelihood ratios, and the unadjusted absolute-risk difference were reported for the exploratory SOFA-2 ≥ 12 rule. The four NE-equivalent categories were study-defined, clinically interpretable strata and were not described as prespecified thresholds. All tests were two-sided, and no correction for multiplicity was applied to exploratory comparisons. Analyses used R 4.4.1, survival 3.6–4, pROC 1.19.0.1, and mice 3.19.0.

Exploratory continuous relationships were examined in complete cases using restricted cubic splines with knots at the 5th, 35th, 65th, and 95th percentiles. The SOFA-2 total-score model was adjusted for age, sex, Charlson index, and lactate; the log-transformed first-24-h five-agent NE-equivalent peak burden model was additionally adjusted for non-cardiovascular SOFA-2. Non-linearity was assessed by likelihood ratio comparison with the corresponding linear model, and marginal standardized risks with 95% CIs were obtained by averaging patient-level predictions. For the drug-composition model, apparent calibration and 1,000-bootstrap optimism-corrected calibration intercepts and slopes were also estimated.

A descriptive external calibration assessment applied the fixed logistic mapping fitted in the primary MIMIC-IV landmark cohort with SOFA-2 total score as the sole predictor: logit(p) = −3.399293 + 0.245548 × total score. The mapping was applied without refitting or recalibration. Because the external endpoints were not isomorphic to the MIMIC-IV outcome, calibration estimates were interpreted within each cohort rather than as proof of endpoint-equivalent transportability. SOFA-1 was retained for discrimination comparison only.

As a supplementary benchmark analysis, SOFA-1, SOFA-2, and the cohort-specific Acute Physiology and Chronic Health Evaluation (APACHE) IVa or APACHE II score were compared by paired DeLong tests in the same patient subset within each cohort. In MIMIC-IV, an additional complete-case analysis without imputation compared the SOFA-2 total score, continuous five-agent NE-equivalent burden, and the drug-composition model with a simple clinical reference model comprising age, lactate, and continuous five-agent NE-equivalent burden (Supplementary Table S21).

## 3 Results

### 3.1 Study cohorts

The MIMIC-IV exposure-eligible source cohort included 11,997 patients, with 2,792 reconstructed deaths by day 28 (23.27%). Of these deaths, 105 occurred at or before the 24-h landmark. The primary landmark cohort included 11,892 patients, with 2,687 deaths between the landmark and ICU day 28 (22.60%) (Figure 1). The eICU transportability cohort included 1,528 patients and 441 derived ICU 28-day events, and the independent-center high-risk stress test cohort included 492 patients and 298 deaths by day 28 (Table 1).

**Figure 1:**
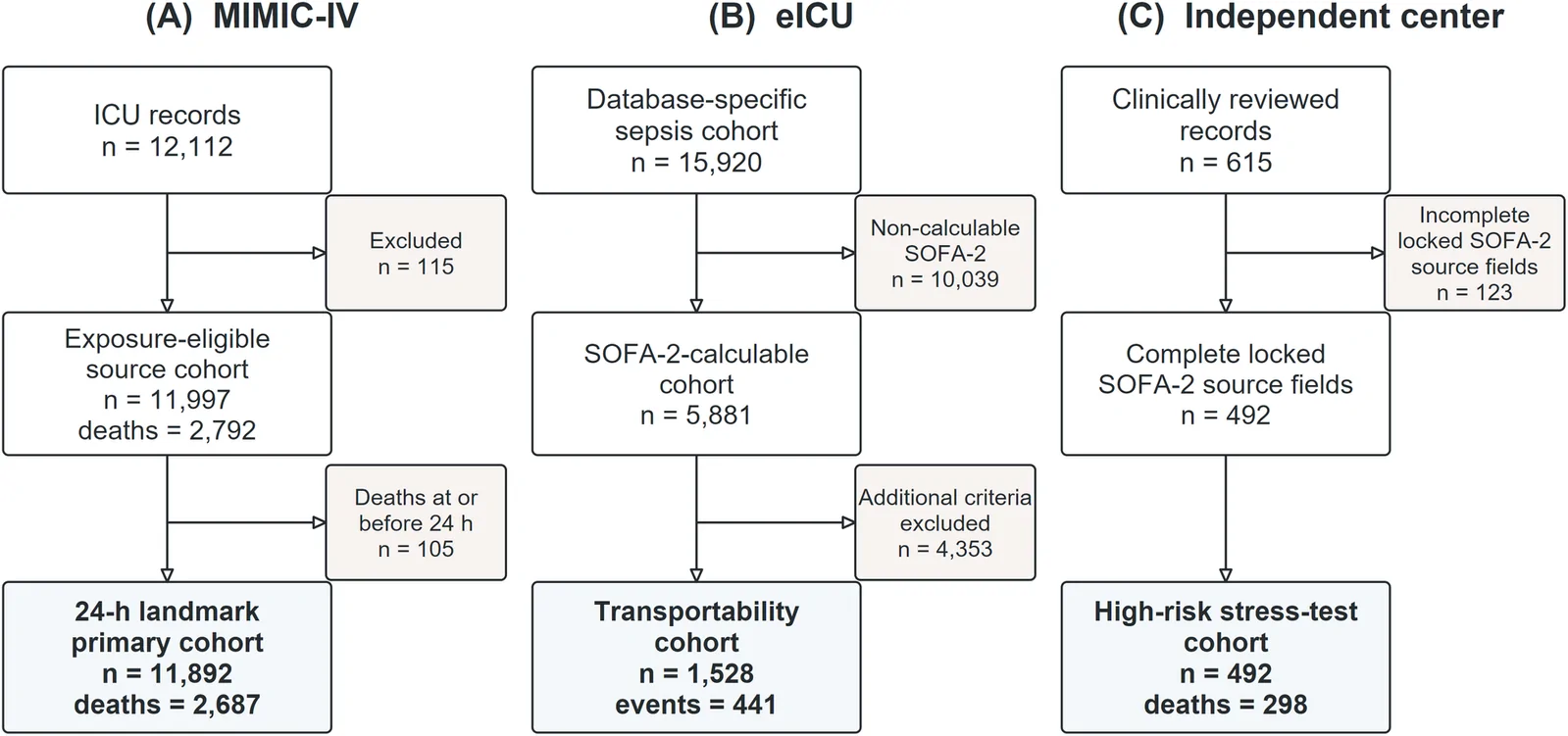
Parallel cohort derivation. **(A)** MIMIC-IV included 12,112 ICU records; 11,997 met exposure eligibility, and exclusion of 105 deaths at or before 24 h left the 11,892-patient landmark primary cohort with 2,687 post-landmark deaths. **(B)** In eICU, 5,881 of 15,920 records were SOFA-2 calculable and 1,528 met the strict transportability criteria. **(C)** At the independent center, 492 of 615 clinically reviewed records had complete locked SOFA-2 source fields; all retained records had first-24-h vasoactive treatment evidence, whereas all 123 exclusions lacked complete source fields and 107 also lacked early vasoactive treatment evidence. Both external cohorts comprised patients alive and in the ICU beyond 24 h and were used for transportability assessment or directional stress testing under cohort-specific endpoints rather than endpoint-equivalent absolute-risk validation.

**Table 1:** Baseline characteristics of the MIMIC-IV 24-h landmark primary cohort and the two external assessment cohorts.

Characteristic	MIMIC-IV 24-h landmark primary cohort	eICU transportability cohort	Independent-center stress test cohort
Number of patients	11,892	1,528	492
Age, years	69.0 [59.0, 79.0]	68.0 [57.0, 78.0]	62.0 [53.0, 73.0]
Male sex	7,157/11,892 (60.2%)	782/1,528 (51.2%)	225/492 (45.7%)
ICU length of stay, days	3.4 [2.0, 7.1]	4.5 [2.4, 8.4]	7.7 [3.2, 15.7]
Hospital length of stay, days	9.0 [5.4, 16.1]	8.1 [4.2, 13.9]	10.8 [4.8, 21.1]
Comorbidity burden	5.0 [3.0, 7.0]	2.0 [1.0, 3.0]	3.0 [2.0, 4.0]
Infection source: bloodstream/endovascular	574/11,892 (4.8%)	128/1,528 (8.4%)	NA (not separately captured)
Infection source: central nervous system	111/11,892 (0.9%)	16/1,528 (1.0%)	17/492 (3.5%)
Infection source: intra-abdominal/hepatobiliary	1,567/11,892 (13.2%)	161/1,528 (10.5%)	97/492 (19.7%)
Infection source: other or unknown	5,162/11,892 (43.4%)	604/1,528 (39.5%)	94/492 (19.1%)
Infection source: pulmonary	4,204/11,892 (35.4%)	535/1,528 (35.0%)	331/492 (67.3%)
Infection source: skin/soft tissue/bone/joint	715/11,892 (6.0%)	94/1,528 (6.2%)	NA (not separately captured)
Infection source: urinary tract	2,197/11,892 (18.5%)	278/1,528 (18.2%)	101/492 (20.5%)
Acute physiology score (cohort-specific)	Not available	APACHE IVa 91 [72, 113]	APACHE II 32 [26, 38]
SOFA-1 total score	7 [5, 10]	10 [7, 12]	10 [8, 12]
SOFA-2 total score	8 [6, 10]	9 [7, 12]	9 [6, 12]
SOFA-1 cardiovascular score	3 [1, 4]	4 [3, 4]	2 [2, 4]
SOFA-2 cardiovascular score	2 [2, 3]	2 [0, 3]	2 [0, 3]
SOFA-2 respiratory score	2 [0, 3]	2 [1, 3]	1 [0, 2]
SOFA-2 neurologic score	0 [0, 1]	2 [1, 3]	4 [2, 4]
SOFA-2 renal score	2 [1, 2]	2 [1, 3]	2 [0, 4]
SOFA-2 hepatic score	0 [0, 0]	0 [0, 1]	0 [0, 1]
SOFA-2 coagulation score	1 [0, 1]	0 [0, 2]	0 [0, 2]
Lactate, mmol/L	2.7 [1.8, 4.3]	2.9 [1.7, 5.5]	2.1 [1.2, 4.2]
White blood cell count, ×10^9^/L	15.6 [11.5, 20.9]	19.7 [13.9, 27.8]	11.4 [7.3, 16.8]
PaO_2_/FiO_2_	150 [88, 240]	236 [162, 336]	260 [173, 364]
Platelet count, ×10^9^/L	147 [104, 207]	156 [93, 232]	172 [90, 268]
Study-defined invasive mechanical ventilation or tracheostomy during the first 24 ICU hours	8,902/11,892 (74.9%)	942/1,528 (61.6%)	457/492 (92.9%)
Study-defined renal replacement therapy during the first 24 ICU hours	854/11,892 (7.2%)	193/1,528 (12.6%)	178/492 (36.2%)
Study-defined vasoactive-drug exposure during the first 24 ICU hours	11,892/11,892 (100.0%)	1,528/1,528 (100.0%)	492/492 (100.0%)
Vasopressin use during the first 24 ICU hours	1,853/11,892 (15.6%)	427/1,528 (27.9%)	Not available
Multiple vasoactive-drug use during the first 24 ICU hours	3,662/11,892 (30.8%)	566/1,528 (37.0%)	89/492 (18.1%)
Primary endpoint	Post-landmark 28-day all-cause mortality through ICU day 28	Database-derived ICU 28-day endpoint	Traceable 28-day all-cause mortality
Primary endpoint events	2,687/11,892 (22.60%)	441/1,528 (28.86%)	298/492 (60.57%)

Continuous variables are presented as median [interquartile range], and categorical variables as events/available denominator (%). For MIMIC-IV, Table 1 reports the 24-h landmark primary cohort (*N* = 11,892), including its infection source composition. Infection sources are non-mutually exclusive, and a patient may contribute to more than one category; other or unknown denotes the absence of a named infection source indicator. Supplementary Table S15A separately reports the exposure-eligible source cohort (*N* = 11,997), while Supplementary Table S15B documents covariate availability; these denominators and purposes are not interchangeable. The MIMIC-IV column directly describes the 24-h landmark primary cohort (11,892 patients; 2,687 post-landmark deaths). The 11,997 patient exposure-eligible source cohort and endpoint lineage are retained in Supplementary Tables S1–S3. Landmark infection source counts were linked by stay_id from the locked descriptive source indicators and were not entered into adjusted models. MIMIC-IV used reconstructed 28-day all-cause mortality; eICU used a database-derived ICU endpoint; the independent center used traceable 28-day all-cause mortality. Acute physiology scores are cohort-specific descriptive measures and should not be interpreted as a harmonized scale across databases. SOFA-2 analyses used locked domain scores aligned with the JAMA 2025 framework. Six-domain arithmetic consistency was verified in all cohorts. Event-level reproducibility was cohort-specific: MIMIC-IV retained executable local SQL/QC, the independent center supported partial retained field reconstruction, and the current eICU cohort did not retain a matching raw event pathway. In MIMIC-IV, mechanical ventilation was defined as invasive mechanical ventilation or tracheostomy during the first 24 ICU hours; non-invasive ventilation, high-flow nasal oxygen, and conventional supplemental oxygen were excluded. Assessable first-day GCS was missing in 29.99% of the MIMIC-IV exposure-eligible source cohort; therefore, derived GCS values are not displayed in the main table. The full source cohort variable completeness assessment is provided in Supplementary Table S3. The composition of comorbidity burden and acute physiology scores differs across cohorts and is reported descriptively only. NA indicates that the infection source category was not separately captured in that cohort and must not be interpreted as 0. In the independent-center cohort, this applies to bloodstream/endovascular infection and skin/soft tissue/bone/joint infection. The complete first-24-h five-agent norepinephrine-equivalent peak burden could be reconstructed reliably only in MIMIC-IV and is therefore reported in the dedicated burden analyses.

All three cohorts comprised patients with sepsis who received early vasoactive therapy, but baseline severity and organ support burden differed. Median SOFA-2 total scores were 8 [6, 10], 9 [7, 12], and 9 [6, 12] in MIMIC-IV, eICU, and the independent center, respectively. Invasive mechanical ventilation or tracheostomy during the first 24 h was used in 74.9%, 61.6%, and 92.9% of patients. Pulmonary infection was recorded in 35.4%, 35.0%, and 67.3%; urinary tract infection in 18.5%, 18.2%, and 20.5%; and intra-abdominal or hepatobiliary infection in 13.2%, 10.5%, and 19.7%, respectively. For MIMIC-IV, Table 1 describes infection sources in the 24-h landmark primary cohort (*N* = 11,892). Supplementary Table S15A retains the infection source distribution in the exposure-eligible source cohort (*N* = 11,997), whereas Supplementary Table S15B reports the availability of infection source covariates in the current landmark modeling file; these denominators and purposes are not interchangeable. Infection source categories were non-mutually exclusive. The independent center did not separately collect bloodstream/endovascular or skin/soft-tissue/bone/joint infection; these cells are shown as NA and should not be interpreted as 0. Supplementary Table S23 reports ICU type, admission source, and administrative admission type only for the MIMIC-IV exposure-eligible source cohort and the ≥60-min duration-qualified source cohort; it does not provide corresponding data for eICU or the independent center. Reliable fields for direct surgical admission and postoperative status were unavailable, and these features were not inferred from ICU names or administrative labels.

The strict eICU transportability cohort was derived from a parent cohort of 15,920 records. SOFA-2 could be calculated in 5,881 records, and 1,528 patients from 99 hospitals remained after screening for a structured sepsis diagnosis, organ dysfunction, vasoactive drug exposure, and complete scores. Compared with records that did not enter the strict cohort, included patients showed large standardized differences in original SOFA score, APACHE IVa score, database-native mechanical- ventilation and vasoactive drug indicators, and hospital mortality (absolute standardized mean differences 1.506, 0.965, 0.779, 0.993, and 0.492, respectively; Supplementary Table S18). These database-native treatment indicators were not harmonized first-24-h study variables and were used only to describe cohort selection.

The independent-center cohort had 298 deaths among 492 patients (28-day all-cause mortality, 60.57%) and a distinctly high-risk case mix with substantial organ support burden: median age 62 [53, 73] years, SOFA-2 total score 9 [6, 12], APACHE II score 32 [26, 38], and lactate 2.1 [1.2, 4.2] mmol/L; 92.9% received invasive mechanical ventilation or tracheostomy during the first 24 h, 36.2% received renal replacement therapy (RRT), and 67.3% had pulmonary infection (Table 1). In the parent sample of 615 records, 322 patients died by day 28 (52.36%); 24 of the 123 excluded patients died (19.51%). This difference indicates that the locked inclusion criteria selectively enriched the cohort for high-risk cases but does not identify the causal source of the mortality difference. The locked data did not contain reliable information on ICU specialty, admission source, referral background, continuous enrollment logs, or postoperative status. We therefore could not confirm consecutive enrollment or exclude selective inclusion. The observed mortality of 60.57% cannot be attributed to these unmeasured factors and does not estimate mortality in the center's general ICU population. This cohort was used only to stress test the direction of the fixed rule in a high-risk case mix, not to extrapolate absolute risk.

In the MIMIC-IV exposure-eligible source cohort, patients who died were older than survivors (72 [61, 82] vs. 68 [58, 77] years), had higher Charlson comorbidity index values (6 [4, 9] vs. 5 [3, 7]), and had higher lactate concentrations [3.5 [2.0, 6.8] vs. 2.5 [1.8, 3.8] mmol/L). Median first-24-h five-agent NE-equivalent peak burden was 0.3 [0.1, 0.6] μg/kg/min among patients who died and 0.1 [0.1, 0.2] μg/kg/min among survivors. Vasopressin use was 32.31% and 11.12%, and multiple vasoactive drug use was 46.31% and 26.65%, respectively (Supplementary Table S2).

### 3.2 Distribution and discrimination of SOFA-1 and SOFA-2 total scores

In the MIMIC-IV 24-h landmark cohort, AUROCs were 0.732 (95% CI 0.721–0.742) for SOFA-1 and 0.731 (95% CI 0.720–0.742) for SOFA-2. The SOFA-2-minus-SOFA-1 AUROC difference was −0.0005 (95% CI −0.0086 to 0.0076; paired DeLong *P* = 0.908), showing no observed overall discrimination advantage for SOFA-2. No equivalence or non-inferiority margin was prespecified, so this result does not establish formal statistical equivalence. AUROCs for the SOFA-1 and SOFA-2 cardiovascular scores were 0.676 and 0.663, respectively (*P* = 0.006) (Table 2; Figure 2; Supplementary Figure S2; Supplementary Table S7).

**Table 2:** Discrimination and internal validation in the MIMIC-IV 24-h landmark cohort.

Model	N	Events	AUROC (95% CI)	Brier score	Comparator	ΔAUROC	DeLong *P*	Corrected AUROC	Corrected Brier	Validation scope
SOFA-1 total	11,892	2,687	0.732 (0.721–0.742)	0.152	—	—	—	—	—	Discrimination
SOFA-2 total	11,892	2,687	0.731 (0.720–0.742)	0.151	SOFA-1 total	−0.0005	0.908	0.731	0.152	1,000-bootstrap internal validation
SOFA-1 cardiovascular	11,892	2,687	0.676 (0.666–0.686)	0.162	—	—	—	—	—	Discrimination
SOFA-2 cardiovascular	11,892	2,687	0.663 (0.652–0.674)	0.162	SOFA-1 cardiovascular	−0.013	0.006	—	—	Discrimination
Non-cardiovascular SOFA-2 (M0)	11,890	2,686	0.708 (0.696–0.719)	0.155	—	—	—	—	—	Development subset
M0 + cardiovascular + continuous burden (M3)	11,890	2,686	0.743 (0.732–0.754)	0.150	M0	+0.035	<0.001	—	—	Development subset
M0 + five drug indicators (M4)	11,890	2,686	0.763 (0.753–0.773)	0.147	M0	+0.055	<0.001	—	—	Development subset
SOFA-2 total + five drug indicators	11,890	2,686	0.768 (0.758–0.778)	0.146	SOFA-2 total	+0.037	<0.001	0.767	0.146	1,000-bootstrap internal validation

The outcome was post-landmark 28-day all-cause mortality. Models involving continuous first-24-h five-agent NE-equivalent peak burden used *N* = 11,890 because two values were unavailable. DeLong methods compare paired AUROCs against the stated comparator. For SOFA-2 vs. SOFA-1 total scores, the SOFA-2-minus-SOFA-1 difference was −0.0005 (95% CI −0.0086 to 0.0076). No equivalence or non-inferiority margin was prespecified. Five-agent norepinephrine-equivalent peak burden was defined as the maximum first-24-h combined value of norepinephrine + epinephrine + dopamine/100 + phenylephrine/10 + vasopressin (U/min) × 2.5. The catecholamine-only sensitivity burden excludes vasopressin conversion. The vasoactive drug-composition model included SOFA-2 total score and five individual drug use indicators; drug class count and multiple drug use were excluded because of deterministic redundancy. The SOFA-2 total-score model underwent 1,000-resample bootstrap internal validation in the full 11,892-patient landmark cohort (seed 20260824). The vasoactive drug-composition model was validated in the 11,890-patient common prediction subset; other incremental models are reported as apparent development sample performance. An em dash indicates not applicable or not performed.

**Figure 2:**
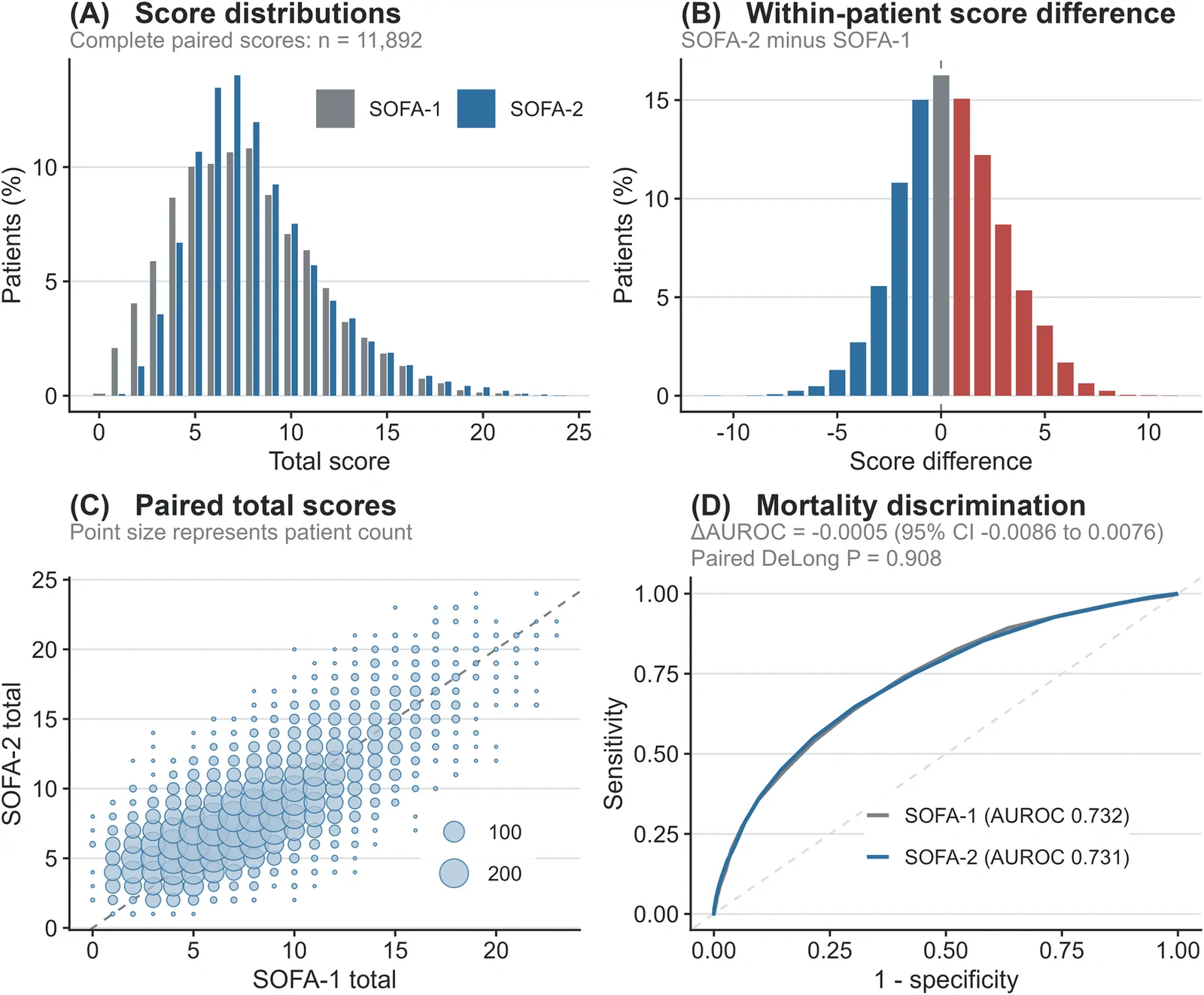
Paired SOFA-1 and SOFA-2 scores and mortality discrimination in the MIMIC-IV 24-h landmark cohort. **(A–C)** show score distributions and paired within-patient comparisons; **(D)** shows ROC curves for post-landmark 28-day all-cause mortality. AUROCs were 0.732 (95% CI 0.721–0.742) for SOFA-1 and 0.731 (95% CI 0.720–0.742) for SOFA-2. The SOFA-2-minus-SOFA-1 difference was −0.0005 (95% CI −0.0086 to 0.0076; paired DeLong *P* = 0.908), showing no observed overall advantage but not establishing formal equivalence. Point size represents the number of patients at each coordinate; the dashed line indicates identical scores. All panels use the MIMIC-IV 24-h landmark cohort (*N* = 11,892; 2,687 events).

### 3.3 Data-derived SOFA-2 stratification and mortality risk

In the complete-case spline analysis of 10,714 patients and 2,464 events, standardized mortality increased smoothly with the SOFA-2 total score. There was no evidence of departure from a linear score effect (*P* for non-linearity = 0.150) and no clear discontinuity at 12, supporting interpretation of ≥12 as an exploratory grouping rather than a biological or treatment threshold (Supplementary Figure S7A).

The data-derived SOFA-2 rule of ≥12 classified 1,879 of 11,892 patients (15.80%) as high risk. Mortality was 17.09% (1,711/10,013) among patients with scores <12 and 51.94% (976/1,879) among those with scores ≥12. The unadjusted risk difference was 34.85 percentage points (95% CI 32.47–37.22), and the multiply imputed adjusted OR was 3.89 (95% CI 3.44–4.39; *P* < 0.001) (Table 3; Figure 3).

**Table 3:** Associations with post-landmark 28-day all-cause mortality in MIMIC-IV.

Analysis block	Exposure	Group	N/events	Observed mortality (%)	Adjusted OR (95% CI)	Standardized risk (%)	Adjusted RD vs. reference (95% CI)	*P*-value
Panel A. SOFA-2 total-score stratum	SOFA-2 total-score stratum	<12	10,013/1,711	17.09	1.00 (reference)	18.31	Reference	—
Panel A. SOFA-2 total-score stratum	SOFA-2 total-score stratum	≥12	1,879/976	51.94	3.89 (3.44–4.39)	43.04	24.73 pp (22.22–27.23)	<0.001
Panel B. SOFA-2 cardiovascular-score stratum	SOFA-2 cardiovascular-score stratum	0–2	7,389/1,125	15.23	1.00 (reference)	18.77	Reference	—
Panel B. SOFA-2 cardiovascular-score stratum	SOFA-2 cardiovascular-score stratum	3–4	4,503/1,562	34.69	1.82 (1.64–2.01)	27.59	8.81 pp (7.27–10.35)	<0.001
Panel C. Five-agent NE-equivalent burden	Peak burden, μg/kg/min	≤0.1	3,753/424	11.30	1.00 (reference)	15.05 (13.84–16.26)	Reference	—
Panel C. Five-agent NE-equivalent burden	Peak burden, μg/kg/min	>0.1–0.3	5,023/974	19.39	1.57 (1.38–1.79)	20.75 (19.69–21.81)	5.70 pp (4.11–7.28)	<0.001
Panel C. Five-agent NE-equivalent burden	Peak burden, μg/kg/min	>0.3–0.5	1,449/498	34.37	2.68 (2.28–3.15)	29.17 (27.09–31.25)	14.12 pp (11.68–16.56)	<0.001
Panel C. Five-agent NE-equivalent burden	Peak burden, μg/kg/min	>0.5	1,665/790	47.45	3.60 (3.07–4.23)	34.51 (32.32–36.69)	19.46 pp (16.86–22.05)	<0.001

SOFA-2 ≥ 12 was derived in the 24-h landmark cohort; ≥11 was retained only as an adjacent-cutpoint sensitivity analysis. Primary adjusted models used 20 multiply imputed datasets. The total-score rule model adjusted for age, sex, Charlson comorbidity index and lactate; cardiovascular-score and norepinephrine-equivalent models additionally adjusted for non-cardiovascular SOFA-2 to avoid part–whole adjustment. Five-agent norepinephrine-equivalent peak burden was the maximum combined burden during the first 24 ICU hours. The four norepinephrine-equivalent peak burden categories were study-defined dose categories used for descriptive presentation and categorical association analyses. They should not be interpreted as optimal, universal clinical, biological, or treatment thresholds. Incremental prediction analyses used continuous first-24-h five-agent norepinephrine-equivalent peak burden. Infection source distributions and covariate availability assessment, non-cardiovascular SOFA-2 adjustment, and complete-case sensitivity analyses are reported in Supplementary Tables S15, S17, S20, respectively. The adjusted OR for each reference row is 1.00. Standardized risks and adjusted risk differences were pooled across 20 imputed datasets using Rubin's rules with delta method variances. An em dash indicates not applicable. All associations are observational and must not be interpreted as treatment effects of vasoactive drugs.

**Figure 3:**
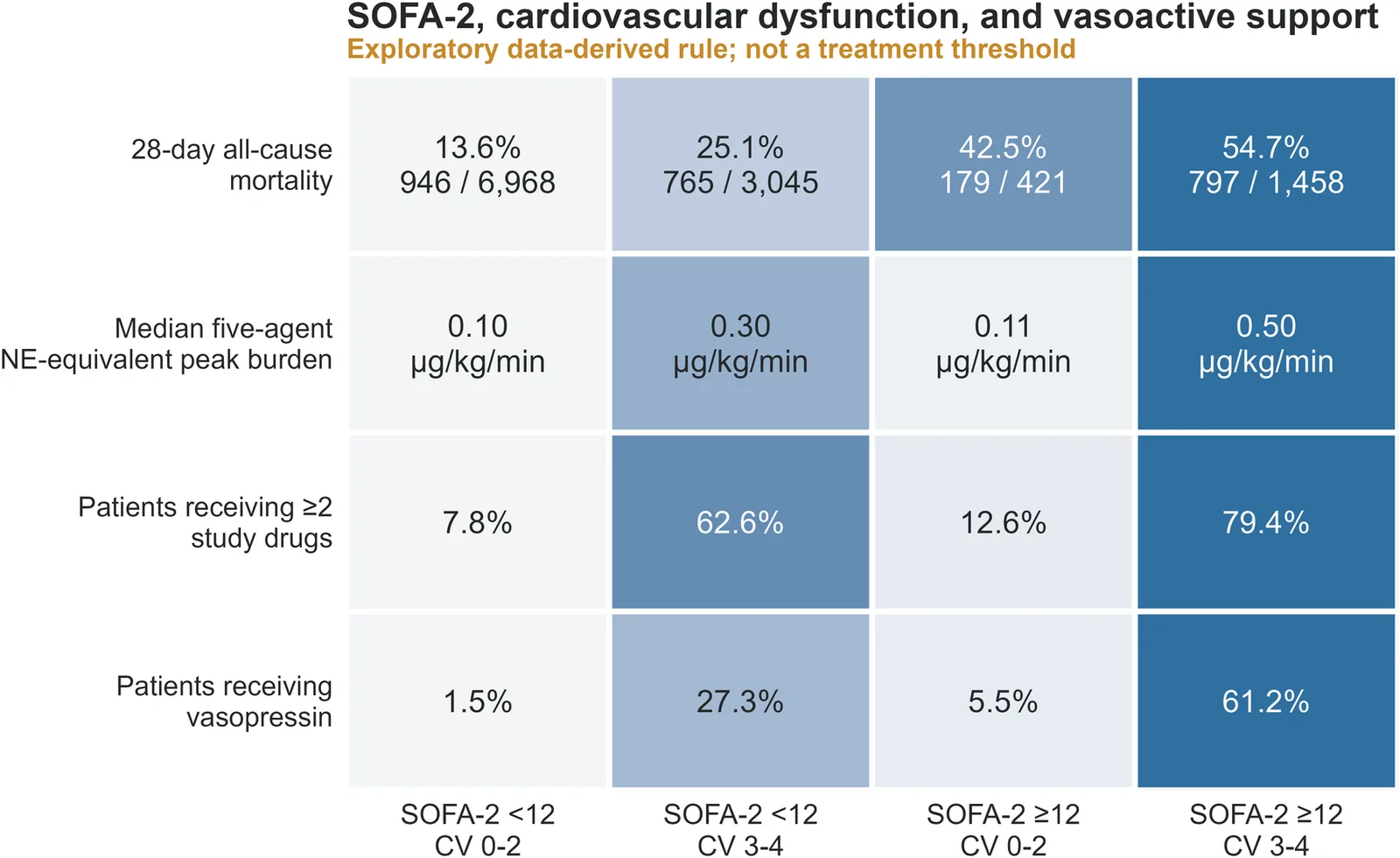
Cross-classification of the data-derived SOFA-2 total-score rule and cardiovascular score in the MIMIC-IV 24-h landmark cohort. Columns define the four total-score (<12/≥12) and cardiovascular-score (0–2/3–4) strata. The first row reports post-landmark 28-day all-cause deaths/N (%). The next three rows report the median first-24-h five-agent norepinephrine-equivalent peak burden (μg/kg/min), the proportion receiving at least two of the five study drugs, and the proportion receiving vasopressin, respectively. Mortality and drug-use indicators used all 11,892 patients (2,687 deaths); peak burden summaries used 11,890 patients (2,686 deaths) because two values were unavailable. The ≥12 rule is data-derived and exploratory and is not a standalone treatment threshold.

Marginal standardized mortality probabilities were 18.31% and 43.04%, yielding an adjusted risk difference of 24.73 percentage points (95% CI 22.22–27.23). Sensitivity was 36.32%, specificity 90.19%, positive predictive value 51.94%, negative predictive value 82.91%, positive likelihood ratio 3.70, and negative likelihood ratio 0.706. Across 1,000 bootstrap samples, ≥12 and ≥11 were selected in 65.4% and 28.7% of cases, respectively.

Mortality was 15.23% (1,125/7,389) for SOFA-2 cardiovascular scores of 0–2 and 34.69% (1,562/4,503) for scores of 3–4. After adjustment for age, sex, Charlson index, lactate, and non-cardiovascular SOFA-2, the OR was 1.82 (95% CI 1.64–2.01). Marginal standardized mortality probabilities were 18.77% and 27.59%, with an adjusted risk difference of 8.81 percentage points (95% CI 7.27–10.35).

Mortality across the cross-classified total-score and cardiovascular-score strata was 13.58% for <12/0–2, 25.12% for <12/3–4, 42.52% for ≥12/0–2, and 54.66% for ≥12/3–4 (Figure 3). This cross-classification describes risk and support patterns and does not define biological phenotypes.

With the adjacent fixed cutpoint of ≥11, the <11 group comprised 9,334 patients with 1,474 deaths (15.79%), and the ≥11 group comprised 2,558 patients with 1,213 deaths (47.42%). The direction of risk separation was preserved. Results for the adjacent-cutpoint sensitivity analysis and threshold performance are summarized in Supplementary Table S14. A score of ≥11 was no longer the optimal cutpoint in the current primary landmark analysis.

### 3.4 Five-agent norepinephrine-equivalent peak burden and mortality risk

The four study-defined five-agent NE-equivalent peak burden categories of ≤0.1, >0.1–0.3, >0.3–0.5, and >0.5 μg/kg/min included 3,753, 5,023, 1,449, and 1,665 patients, with mortality rates of 11.30%, 19.39%, 34.37%, and 47.45%, respectively (Figure 4B). With ≤0.1 μg/kg/min as the reference, ORs adjusted for age, sex, Charlson index, lactate, and non-cardiovascular SOFA-2 were 1.57 (95% CI 1.38–1.79), 2.68 (2.28–3.15), and 3.60 (3.07–4.23); the corresponding adjusted risk differences were 5.70, 14.12, and 19.46 percentage points (Table 3; Figure 4C; Supplementary Table S10).

**Figure 4:**
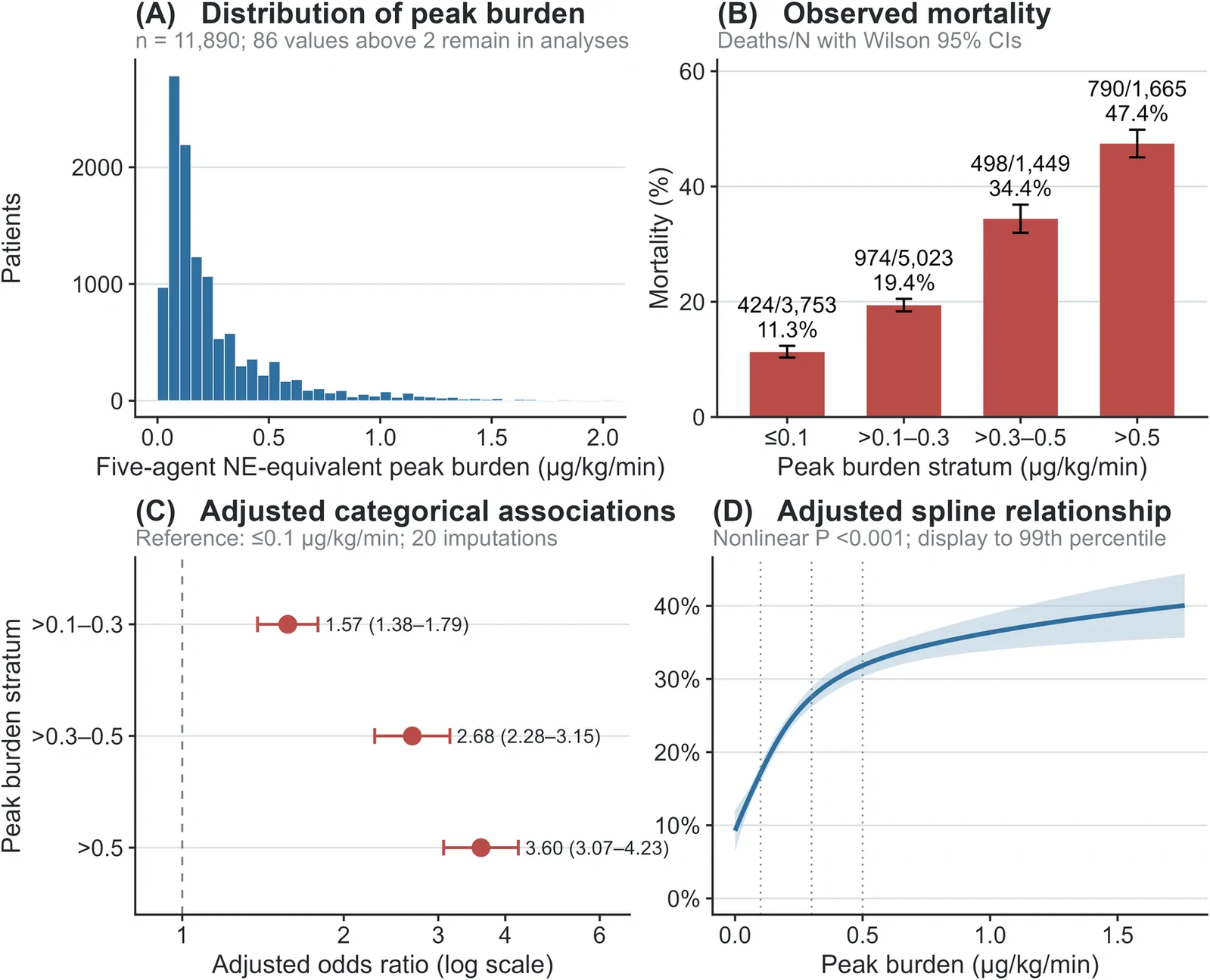
First-24-h five-agent norepinephrine-equivalent peak burden and post-landmark 28-day all-cause mortality in MIMIC-IV. Analyses with finite burden included 11,890 patients and 2,686 events. **(A)** The distribution is displayed to 2 μg/kg/min; 86 higher values remain in all analyses. **(B)** Bars report deaths/N and observed mortality with Wilson 95% CIs. **(C)** Points report adjusted ORs and 95% CIs for the three study-defined categories relative to ≤0.1 μg/kg/min, pooled across 20 imputed datasets after adjustment for age, sex, Charlson index, lactate, and non-cardiovascular SOFA-2. **(D)** The restricted cubic spline shows marginal standardized mortality after the same covariate adjustment in complete cases; the curve is displayed to the 99th percentile, shading denotes 95% CI, dotted lines mark the descriptive category boundaries, and *P* for non-linearity was <0.001. Categories and curves describe prognostic association and are not treatment thresholds.

In the complete-case continuous analysis of 10,713 patients and 2,463 events, the log-scaled restricted cubic spline showed a graded non-linear relationship between peak burden and standardized mortality (*P* for non-linearity < 0.001), with the steepest increase at lower burden values and no empirical support for a single universal dose threshold (Figure 4D; Supplementary Figure S7B).

In the MIMIC-IV 24-h landmark cohort, the Spearman correlation between the SOFA-2 cardiovascular score and continuous five-agent NE-equivalent burden was *ρ* = 0.727; Cramér's V for the cardiovascular score and the four-level peak burden categories was 0.492 (Supplementary Figure S4; Supplementary Table S16). Patients with a cardiovascular score of 3–4 but peak burden ≤0.1 accounted for 4.8%, whereas 8.5% had burden >0.5 with a cardiovascular score of 0–2. Thus, the two measures captured overlapping but non-identical information about support intensity.

In the MIMIC-IV exposure-eligible source cohort, positive-rate continuous infusions of dobutamine and milrinone during the first 24 h were recorded for 300/11,997 (2.5%) and 344/11,997 (2.9%) patients, respectively. At least one infusion episode lasting ≥60 min was present in 265 (2.2%) and 336 (2.8%) patients. Under the SOFA-2 cardiovascular rules used in this study, both drugs were classified as other vasopressors/inotropes but were excluded from the five-agent NE-equivalent formula. Detailed drug mapping is provided in Supplementary Table S4.

The catecholamine-only NE-equivalent and non-cardiovascular SOFA-2-adjusted sensitivity analyses showed the same risk gradient (Supplementary Figure S3; Supplementary Tables S5, S17). Complete coefficients for the main multivariable models are reported in Supplementary Table S6. These findings make it less likely that the associations arose entirely from vasopressin conversion or overlap with the cardiovascular component of the total score, but they do not remove confounding by indication or residual confounding.

In the duration-qualified sensitivity analysis requiring merged infusion episodes for the same drug to last ≥60 min, the landmark cohort included 10,858 patients and 2,611 deaths. AUROC was 0.723 for both the SOFA-1 and SOFA-2 total scores (DeLong *P* = 0.918), and the associations for the ≥12 stratum, cardiovascular score, and first-24-h five-agent NE-equivalent peak burden were directionally consistent with the primary analysis (Supplementary Table S22).

### 3.5 Model performance after adding vasoactive drug information to SOFA-2

In the same complete-case subset of 11,890 patients and 2,686 events, AUROCs for M0, M1, M2, M3, M4, and M5 were 0.708, 0.739, 0.735, 0.743, 0.763, and 0.731, respectively (Table 2; Supplementary Tables S7, S24).

The exploratory model combining the SOFA-2 total score with five drug use indicators had an apparent AUROC of 0.768 (95% CI 0.758–0.778) and Brier score of 0.146; after 1,000 bootstrap resamples, corrected values were 0.767 and 0.146, respectively. In the development data, the apparent calibration intercept was 0.000, slope 1.000, and observed-to-expected ratio 1.000; bootstrap-corrected intercept and slope were 0.001 and 0.998, respectively. These internal estimates do not establish transportable calibration or clinical deployment readiness (Figure 5; Supplementary Figure S6; Supplementary Table S19).

**Figure 5:**
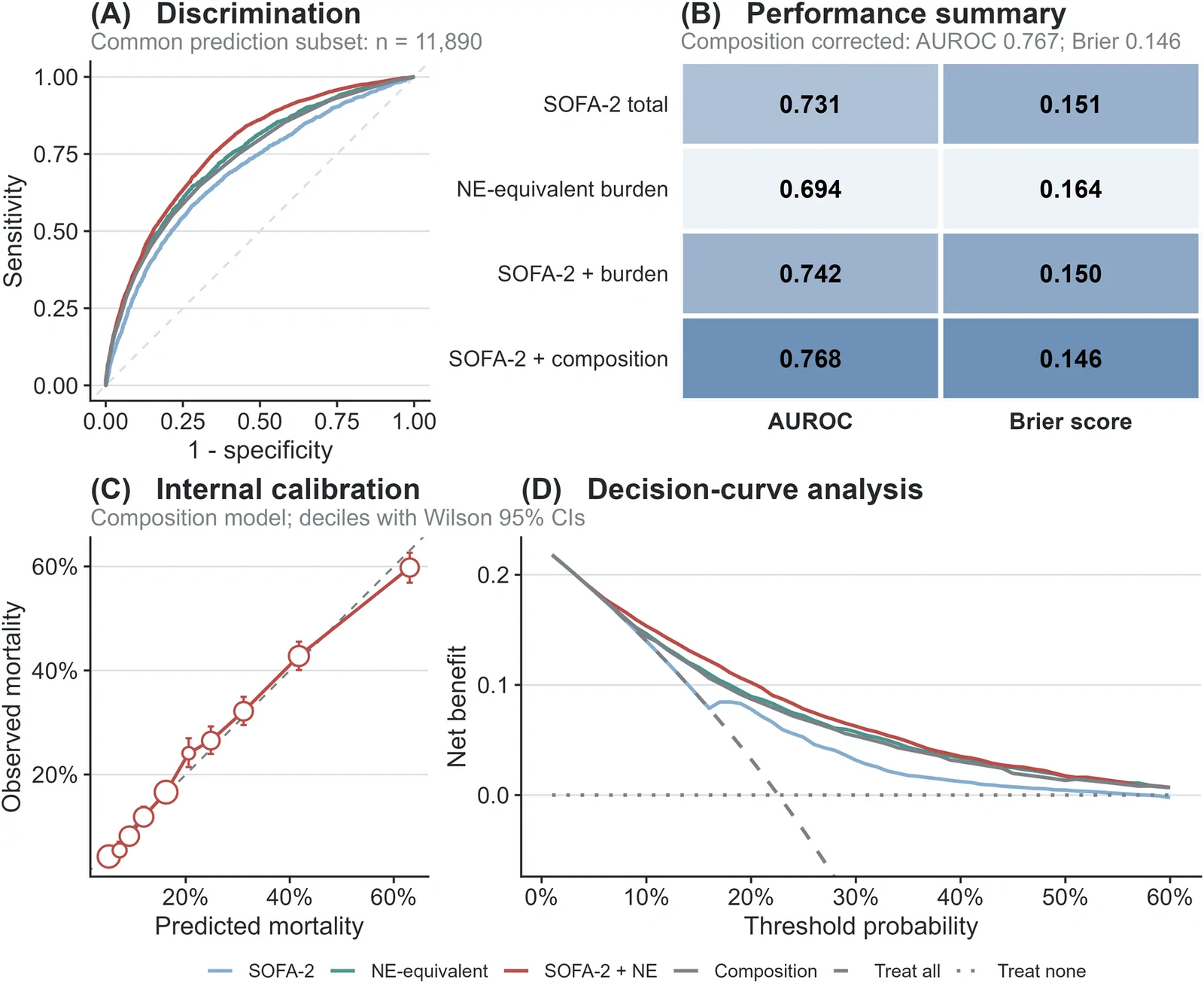
Incremental risk stratification performance in the MIMIC-IV 24-h landmark cohort. **(A)** ROC curves for SOFA-2 total score, continuous first-24-h five-agent norepinephrine-equivalent peak burden, SOFA-2 plus continuous peak burden, and the vasoactive drug-composition model. **(B)** Corresponding AUROCs and Brier scores. **(C)** Calibration of the composition model. **(D)** Decision curves for SOFA-2 total (light blue solid), five-agent norepinephrine-equivalent peak burden (teal solid), SOFA-2 plus peak burden (red solid), SOFA-2 plus vasoactive drug composition (gray solid), Treat all (dark-gray long-dashed), and Treat none (light-gray dotted). The composition model included only SOFA-2 total score and five individual drug use indicators; drug class count and multiple drug use were excluded because of deterministic redundancy. The composition model had an apparent AUROC of 0.768 and Brier score of 0.146; after 1,000 bootstrap resamples, corrected values were 0.767 and 0.146. Calibration points summarize deciles of predicted risk; error bars show Wilson score 95% CIs. Decision curve analysis was performed only as a hypothetical risk stratification scenario in the MIMIC-IV development sample. Threshold probabilities do not correspond to treatment escalation thresholds, and the curves do not compare causal effects of vasoactive strategies. Model coefficients, rank, variance inflation factors (VIFs), and bootstrap results are reported in Supplementary Table S19.

In the MIMIC-IV complete-case benchmark subset of 10,713 patients and 2,463 events, AUROCs for the simple clinical reference model, SOFA-2 total score, and drug-composition model were 0.715, 0.737, and 0.777, respectively. Compared with the simple clinical reference model, the AUROC difference was 0.022 for the SOFA-2 total score (*P* = 0.002) and 0.062 for the composition model (*P* < 0.001) (Supplementary Table S21).

In the common prediction subset, M3 (non-cardiovascular SOFA-2 plus cardiovascular score and continuous first-24-h five-agent NE-equivalent peak burden) had an AUROC of 0.743, compared with 0.739 for M1 (non-cardiovascular SOFA-2 plus cardiovascular score). This descriptive difference of 0.004 was not subjected to a direct prespecified paired test. The drug-composition model retained additional information about the structure of support. These are development sample predictive results and do not establish independent biological effects or clinical decision utility.

The primary multiply imputed results were directionally consistent with complete-case analyses (Supplementary Table S20). Decision curve analysis was framed only as a hypothetical risk stratification scenario at selected threshold probabilities; those probabilities are not treatment thresholds, and the analysis does not compare causal effects of vasoactive strategies (Supplementary Figure S5).

### 3.6 Transportability assessment and high-risk stress testing in heterogeneous external cohorts

In the eICU transportability cohort, AUROCs for the SOFA-1 and SOFA-2 total scores were 0.698 and 0.726, respectively. The hospital-clustered bootstrap AUROC difference was 0.028 (95% CI 0.0004–0.054; *P* = 0.047). Under the fixed ≥12 rule, event rates were 19.45% for scores <12 and 50.11% for scores ≥12, with an OR of 4.16 (95% CI 3.28–5.27). For the fixed SOFA-2 mapping, the descriptive calibration intercept was −0.065, the slope was 0.872, the Brier score was 0.177, and the observed-to-expected ratio was 0.963. These estimates apply to the database-derived ICU endpoint and do not establish endpoint-equivalent risk transport. Fixed-stratum and MIMIC-IV threshold stability results are presented in Supplementary Table S13.

In the independent-center high-risk stress test cohort, AUROCs for the SOFA-1 and SOFA-2 total scores were 0.657 and 0.663, respectively (*P* = 0.745). Under the fixed ≥12 rule, mortality was 54.38% for scores <12 and 73.29% for scores ≥12, with an OR of 2.30 (95% CI 1.53–3.47). The calibration intercept was 1.594, the slope was 0.552, the Brier score was 0.319, and the observed-to-expected ratio was 1.998, indicating that the MIMIC-IV mapping underestimated risk at this center (Tables 4, 5; Figure 6).

**Table 4:** Fixed-rule transportability and high-risk stress testing.

Cohort	Endpoint	N	Events	SOFA-1 AUROC	SOFA-2 AUROC	SOFA-2 < 12 event rate	SOFA-2 ≥ 12 event rate	CV 0–2 event rate	CV 3–4 event rate
eICU transportability cohort	Database-derived ICU 28-day endpoint	1,528	441/1,528 (28.86%)	0.698	0.726	206/1,059 (19.45%)	235/469 (50.11%)	237/1,027 (23.08%)	204/501 (40.72%)
Independent-center stress test cohort	Traceable 28-day all-cause mortality	492	298/492 (60.57%)	0.657	0.663	180/331 (54.38%)	118/161 (73.29%)	190/354 (53.67%)	108/138 (78.26%)

eICU used a database-derived ICU endpoint; the independent center used traceable 28-day all-cause mortality. The SOFA-2 ≥ 12 rule was applied without reestimation. eICU in-hospital mortality, included-vs.-excluded comparisons, and hospital-clustered analyses are reported in Supplementary Table S18. Event rates are presented as events/N (%). The independent center remains anonymous in all submission materials.

CV, cardiovascular.

**Table 5:** Descriptive calibration assessment of the fixed MIMIC-IV landmark SOFA-2 risk mapping.

Cohort	Model	N/events	Calibration intercept	Calibration slope	Brier score	O/E ratio	Interpretation
eICU transportability cohort	SOFA-1 total score	1,528/441	—	—	—	—	Discrimination comparison only; calibration not evaluated
eICU transportability cohort	SOFA-2 total score	1,528/441	−0.065	0.872	0.177	0.963	Approximate calibration for the database-derived ICU endpoint; not proof of endpoint-equivalent transportability
Independent-center stress test cohort	SOFA-1 total score	492/298	—	—	—	—	Discrimination comparison only; calibration not evaluated
Independent-center stress test cohort	SOFA-2 total score	492/298	1.594	0.552	0.319	1.998	Marked risk underprediction

The SOFA-2 mapping was logit(p) = −3.399293 + 0.245548 × SOFA-2 total score and was applied without refitting. Intercept 0, slope 1, and O/E 1 indicate ideal calibration. Estimates are cohort-specific point estimates because aligned uncertainty intervals were not available in the retained analysis outputs.

**Figure 6:**
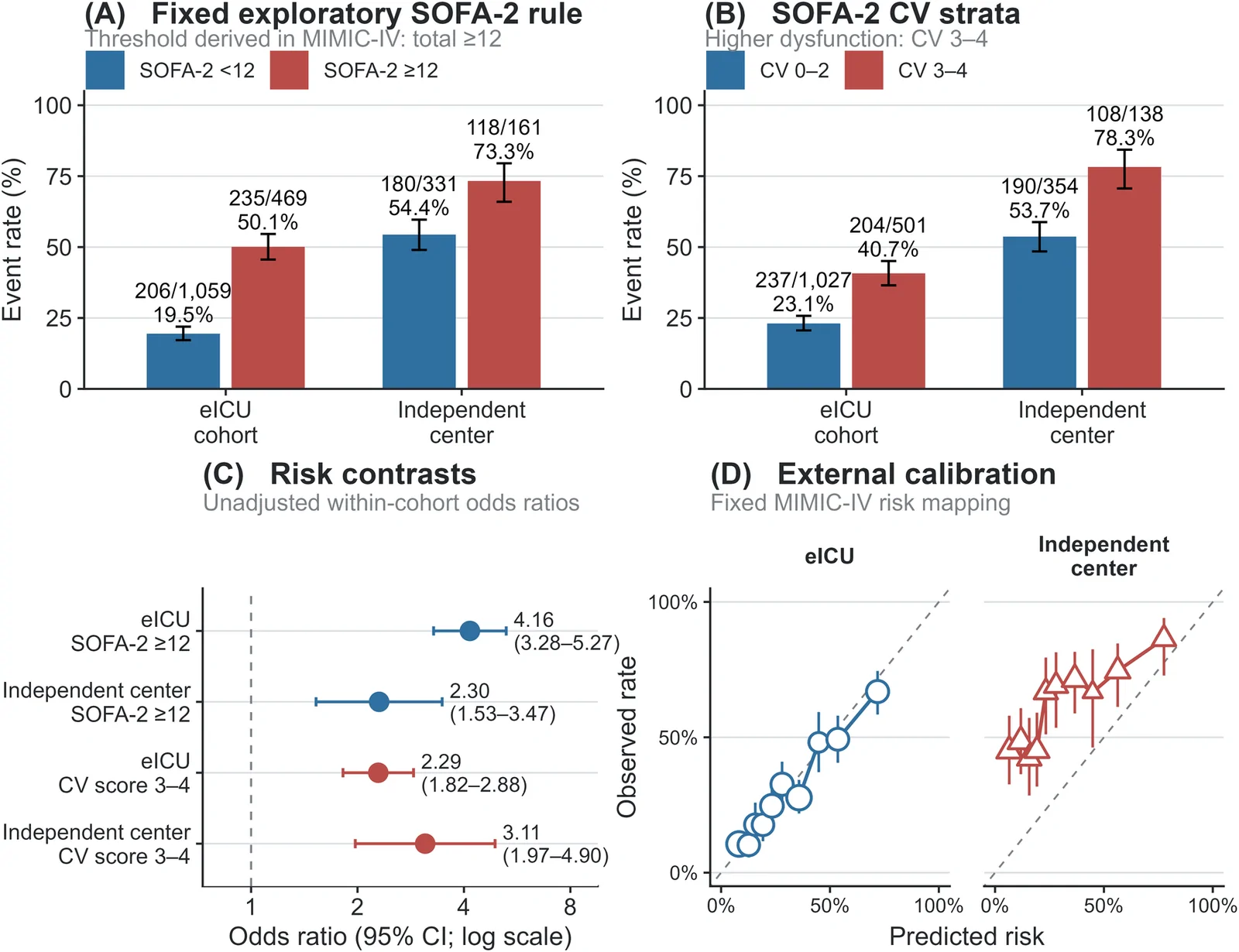
Transportability assessment and high-risk stress testing of the fixed SOFA-2 ≥ 12 rule. **(A)** Fixed total-score strata and **(B)** cardiovascular score strata report cohort-specific events/N and observed event rates; error bars are Wilson score 95% CIs. **(C)** Reports unadjusted within-cohort ORs with 95% CIs. **(D)** External calibration of the fixed MIMIC-IV SOFA-2 logistic mapping in eICU and the independent center; points show observed risk by predicted risk group, error bars are Wilson score 95% CIs, and point size reflects group size. eICU used a database-derived ICU 28-day endpoint, whereas the independent center used traceable 28-day all-cause mortality. Both cohorts required survival and ICU stay beyond 24 h. Neither cutpoints nor model parameters were reestimated; endpoint differences and cohort labels denote inferential roles rather than endpoint-equivalent validation.

Among patients with an available APACHE score, the eICU comparison included 1,392 patients and 417 events. The SOFA-2 total-score AUROC was 0.725, exceeding the APACHE IVa AUROC by 0.039 (*P* = 0.013). The independent-center comparison included 492 patients and 298 events; AUROCs were 0.663 for the SOFA-2 total score and 0.658 for APACHE II (*P* = 0.847) (Supplementary Table S21).

## 4 Discussion

This study had three principal findings. First, SOFA-2 showed no observed overall mortality discrimination advantage over SOFA-1 in patients who received early vasoactive therapy and were alive and still in the ICU beyond 24 h. Second, the SOFA-2 cardiovascular score and first-24-h five-agent NE-equivalent peak burden provided complementary risk description beyond non-cardiovascular organ dysfunction. Third, an exploratory drug-composition model improved development sample discrimination and Brier score, but its external validity and clinical utility remain unestablished.

The absence of an overall AUROC gain may reflect information saturation in this selected landmark cohort. SOFA-1 already integrates six organ systems, and restricting analysis to patients exposed to vasoactive therapy who survived to 24 h may compress between-patient ranking differences. Updates within individual SOFA-2 domains may also be offset when the six domains are summed, and 28-day mortality may be less sensitive than other uses of organ support classification. Recent ICU admission studies reported modest SOFA-2 gains in broader populations (7–9), but differences in population, assessment time, and endpoint limit direct comparison. Because no equivalence or non-inferiority margin was prespecified, our result supports only “no observed advantage,” not formal equivalence or absence of value in other settings.

The SOFA-2 cardiovascular score also had lower standalone discrimination than its SOFA-1 counterpart. The revised domain emphasizes support level, additional vasoactive or inotropic agents, and mechanical circulatory support; these changes need not improve monotonic ranking for a particular mortality endpoint in every selected population. This finding argues against implying that the SOFA-2 cardiovascular domain is uniformly superior, while leaving open its value for describing contemporary support states.

The cardiovascular score and first-24-h five-agent NE-equivalent peak burden measure related but non-identical constructs. The score is a discrete description of circulatory support level that can incorporate mean arterial pressure, other inotropes, and mechanical support; peak burden is a continuous summary of observed combined drug intensity that retains dose resolution but omits duration and trajectory. Discordant classifications are therefore expected. Their complementarity is methodologically plausible, but it does not establish independent pathophysiologic pathways. Prior sepsis studies also linked NE-equivalent measures with prognosis (24).

Vasoactive burden and drug composition are joint manifestations of illness severity, treatment response, and clinical decision-making. They may reflect vasoplegia, myocardial dysfunction, persistent hypoperfusion, treatment resistance, local practice, treatment goals, or unmeasured limitations of care. Adjustment for non-cardiovascular SOFA-2 reduces part–whole conditioning but cannot remove confounding by indication. Individual coefficients in the composition model therefore do not represent comparative drug efficacy, protection, or harm, and changing a drug or dose cannot be inferred to improve outcome from these data.

The external findings further separate ranking from calibration. Fixed strata preserved the direction of relative risk ranking, but the independent center had markedly higher baseline risk and the MIMIC-IV mapping underestimated absolute risk. This coexistence is expected when case mix and endpoint definitions differ: Discrimination can persist while the calibration intercept, slope, and observed-to-expected ratio shift. External use would require endpoint-aligned multicenter validation and center-specific recalibration rather than direct transfer of MIMIC-IV probabilities.

The practical implication is structured reassessment after risk identification, not automatic treatment escalation. At 24 h, the total score, cardiovascular score, observed drug intensity, and treatment composition may contextualize monitoring, repeated perfusion and organ support review, risk communication, and research enrichment. Future work should evaluate time-weighted dose, peak duration, dose trajectories, shock subtypes, causal methods, and prospective decision pathways in multicenter cohorts with aligned outcomes.

### 4.1 Clinical implications

The findings support research-level multilayered risk description at 24 h after ICU admission. The SOFA-2 total score summarizes overall organ dysfunction, the cardiovascular score reflects the level of circulatory support, and the peak observed five-agent NE-equivalent burden and drug composition describe dose and treatment complexity. These measures may contextualize risk communication and integrated clinical reassessment. This study did not test whether their use changes monitoring decisions, treatment, or patient outcomes.

These measures should complement, rather than replace, bedside assessment. The exploratory ≥12 rule should not mandate an increase in vasopressor dose or a specific drug regimen, and the observed drug associations cannot predict benefit from a particular treatment strategy.

### 4.2 Limitations

This study has several limitations. First, the 24-h conditional landmark excluded patients who died or left the ICU before that point; the findings therefore apply only to patients alive and still in the ICU beyond 24 h and cannot be generalized to all patients receiving early vasoactive therapy. Second, case identification and endpoints were not fully isomorphic, and the eICU endpoint could not capture deaths occurring after ICU discharge through day 28.

Third, event-level reproducibility of SOFA-2 differed across cohorts, particularly for sedation and GCS look-back, urine duration, RRT identification, infusion duration, and mechanical support mapping. Arithmetic checks of locked scores do not establish fully equivalent implementation, limiting cross-database comparability.

Fourth, vasoactive drug exposure is influenced by indication, treatment targets, hemodynamic state, clinician preference, and center practice, so residual confounding is unavoidable. The five-agent conversion is a prespecified operational definition, and conversion factors differ across studies. The first-24-h five-agent NE-equivalent peak burden is not an average dose and does not capture duration, area under the dose curve, escalation rate, or response trajectory.

Fifth, the ≥12 rule was a data-derived exploratory rule with limited bootstrap stability. Sixth, the drug-composition model has undergone internal validation only. Admission date or year was not retained in the locked patient-level landmark file, so a temporal validation split could not be performed without reconstructing the source extraction. Finally, MIMIC-IV, eICU, and the independent center span different health systems and eras (2008–2022, 2014–2015, and 2023–2026), during which coding and treatment practice may have changed.

## 5 Conclusion

Among patients with sepsis who received vasoactive therapy during the first 24 h and were alive and still in the ICU beyond 24 h, SOFA-2 showed no observed overall discrimination advantage over SOFA-1 for 28-day all-cause mortality. The SOFA-2 cardiovascular score and first-24-h five-agent NE-equivalent peak burden provided complementary observational prognostic information alongside overall organ dysfunction, and drug composition indicators improved discrimination in the MIMIC-IV development data. These results support risk communication and structured reassessment at 24 h, not automatic treatment escalation. The ≥12 rule is data-derived and exploratory, and drug-related results do not support causal inference about specific strategies. Endpoint-aligned multicenter validation and recalibration are required before clinical use.

## Data Availability

The MIMIC-IV v3.1 (https://physionet.org/content/mimiciv/3.1/) and eICU-CRD v2.0 (https://physionet.org/content/eicu-crd/2.0/) datasets are available through PhysioNet to credentialed users who complete the required training and sign the applicable data use agreements. De-identified participant-level data from the independent-center cohort may be available from the corresponding author upon reasonable request, subject to approval by the relevant ethics committee and institution.
