# Supplementary material for: Prognostic discrimination of SOFA-2 vs. SOFA-1 and incremental information from cardiovascular scoring and vasoactive support intensity in patients with sepsis receiving early vasoactive therapy
Source: Front Cardiovasc Med. 2026 Sep 30;13:1918792. doi: 10.3389/fcvm.2026.1918792 (PMC13661955; doi:10.3389/fcvm.2026.1918792)
Supplement: Supplementary file 1 [file Datasheet1.docx]

Supplementary Figures S1–S7 and Supplementary Tables S1–S24

Supplementary Figure S1. Missingness in the MIMIC-IV 24-hour landmark cohort. The cohort comprised 11,892 patients who were alive beyond ICU admission +24 hours, including 2,687 post-landmark deaths by ICU day 28. (A) Variable-level missingness: lactate was missing in 1,178/11,892 patients (9.91%), five-agent norepinephrine-equivalent peak burden in 2/11,892 (0.02%), and SOFA-1 total, SOFA-2 total and the 28-day endpoint in 0/11,892. (B) Patient-level number of missing analysis fields: 10,713 patients (90.1%) had no missing field, 1,178 (9.9%) had one and 1 (0.0%) had two. Lactate was imputed by predictive mean matching in 20 datasets for the primary adjusted association models; outcomes, SOFA scores and vasoactive exposures were retained as observed. ICU, intensive care unit; SOFA, Sequential Organ Failure Assessment.

Supplementary Figure S2. Paired SOFA-1 and SOFA-2 total-score distributions at the 24-hour landmark. The analysis included the same 11,892 MIMIC-IV patients who were alive beyond ICU admission +24 hours; 2,687 subsequently died by ICU day 28. (A) Proportion of patients at each integer SOFA-1 and SOFA-2 total score. (B) Distribution of the within-patient score difference, calculated as SOFA-2 minus SOFA-1; negative values indicate a lower SOFA-2 score, zero indicates no change and positive values indicate a higher SOFA-2 score. ICU, intensive care unit; SOFA, Sequential Organ Failure Assessment.

Supplementary Figure S3. Catecholamine-only norepinephrine-equivalent sensitivity analysis in the MIMIC-IV 24-hour landmark cohort (N = 11,890; 2,686 post-landmark deaths). Time zero was ICU admission +24 hours. (A) Observed post-landmark 28-day all-cause mortality by catecholamine-only norepinephrine-equivalent burden, with Wilson score 95% confidence intervals. (B) Adjusted odds ratios and 95% confidence intervals pooled across 20 multiply imputed datasets; the ≤0.1 μg/kg/min group was the reference. Models were adjusted for age, sex, Charlson comorbidity index, lactate, and non-cardiovascular SOFA-2. These associations are prognostic and do not estimate causal effects of drug selection or dose. CI, confidence interval; ICU, intensive care unit; NE, norepinephrine; OR, odds ratio; SOFA, Sequential Organ Failure Assessment.

Supplementary Figure S4. Vasoactive-drug composition across study-defined support-intensity strata. The analysis included 11,890 MIMIC-IV patients with finite first-24-hour five-agent norepinephrine-equivalent peak burden, including 2,686 post-landmark deaths by ICU day 28. Columns show the four study-defined burden categories (≤0.1, >0.1–0.3, >0.3–0.5 and >0.5 μg/kg/min); cells report n/N (%) within each category. The five-agent burden was calculated as norepinephrine + epinephrine + dopamine/100 + phenylephrine/10 + vasopressin (U/min) ×2.5. Drug indicators are non-mutually exclusive; multi-vasopressor use denotes use of at least two of the five drugs during the first 24 hours. ICU, intensive care unit.

Supplementary Figure S5. Decision-curve analysis for a hypothetical post-landmark risk-stratification scenario in the MIMIC-IV common prediction subset. Curves were evaluated in 11,890 patients alive beyond ICU admission +24 hours with complete prediction variables, including 2,686 deaths by ICU day 28. The displayed logistic models use SOFA-2 total, continuous five-agent norepinephrine-equivalent peak burden, SOFA-2 total plus continuous burden, or SOFA-2 total plus five first-24-hour drug indicators. Net benefit is shown across threshold probabilities relative to treat-all and treat-none. The thresholds are hypothetical risk-classification preferences and are not treatment-escalation thresholds. This internally evaluated development-sample analysis was not externally validated and does not establish a causal treatment strategy.

Supplementary Figure S6. Internal calibration and bootstrap-corrected performance in the MIMIC-IV common prediction subset (N=11,890; 2,686 events). (A) Observed mortality is plotted against mean predicted mortality for the model containing SOFA-2 total and five first-24-hour drug indicators; the diagonal denotes perfect agreement, circle size reflects group size, and error bars are Wilson 95% CIs. Apparent development-sample calibration intercept, slope, and observed-to-expected ratio were 0.000, 1.000, and 1.000. (B) After 1,000 bootstrap resamples, corrected AUROCs were 0.731 for SOFA-2 total and 0.767 for the composition model; corrected Brier scores were 0.151 and 0.146. For the composition model, the bootstrap-corrected calibration intercept and slope were 0.001 and 0.998. These internal estimates do not establish transportable calibration or a deployable calculator.

Supplementary Figure S7. Continuous adjusted relationships of SOFA-2 total score and first-24-hour five-agent norepinephrine-equivalent peak burden with post-landmark 28-day all-cause mortality in MIMIC-IV. (A) The complete-case SOFA-2 analysis included 10,714 patients and 2,464 events and adjusted for age, sex, Charlson index, and lactate. Knots were at scores 4, 7, 9, and 15; P for nonlinearity was 0.150. The dashed line marks the exploratory ≥12 rule and does not indicate a risk discontinuity or treatment threshold. (B) The complete-case burden analysis included 10,713 patients and 2,463 events and additionally adjusted for non-cardiovascular SOFA-2. Burden was transformed as log[1+burden/0.1], with knots corresponding to 0.030, 0.100, 0.220, and 0.949 μg/kg/min; P for nonlinearity was <0.001. The display is limited to the 99th percentile, and dotted lines mark the four descriptive category boundaries. Curves show marginal standardized mortality and shaded 95% CIs. These exploratory associations do not define universal dose or treatment thresholds.

Supplementary Table S1. Cohort definition, selection, and endpoint summary

| **Cohort** | **Role** | **Operational definition** | **N** | **Events** | **Study endpoint events** |
| --- | --- | --- | --- | --- | --- |
| MIMIC-IV exposure-eligible source cohort | Endpoint lineage | Official mimiciv_derived.sepsis3 cohort; suspected infection plus SOFA increase ≥2 | 11,997 | 2,792 | 2,792/11,997 (23.27%) |
| MIMIC-IV 24-hour landmark cohort | Primary analysis | Source-cohort patients strictly alive beyond ICU admission +24 h; follow-up through ICU day 28 | 11,892 | 2,687 | 2,687/11,892 (22.60%) |
| eICU transportability cohort | Transportability assessment | Structured sepsis/septic shock diagnosis within ICU admission ±24 h plus complete first‑day SOFA ≥2 The strict cohort required ICU stay and survival beyond 24 hours; fixed strata were assessed at the 24-hour index. | 1,528 | 441 | 441/1,528 (28.86%) |
| Independent-center stress-test cohort | High-risk stress test | Explicit or suspected infection plus SOFA increase ≥2, with final clinician adjudication; 615 records were clinically reviewed, and 492 with complete locked SOFA-2 source fields and first-24-hour vasoactive-treatment evidence were retained. The retained cohort had 298/492 deaths (60.57%); the 615-record source cohort had 322 deaths (52.36%), whereas the 123 excluded records had 24 deaths (19.51%), documenting selection enrichment. All exclusions lacked complete locked SOFA-2 source fields, and 107 also lacked early vasoactive-treatment evidence. Eligible ICU admissions were recorded from 1 January 2023 to 1 April 2026; 28-day all-cause mortality was ascertainable for all included patients. All included patients remained in the ICU and were alive beyond 24 hours; fixed strata were assessed at the 24-hour index, and this cohort was used only as a directional stress test. | 492 | 298 | 298/492 (60.57%); traceable 28-day all‑cause mortality |

Note. MIMIC-IV analyses used PhysioNet version 3.1, released on 11 October 2024 (DOI: 10.13026/kpb9-mt58); the database-access certification recorded for this study was dated 25 January 2025 (certificate no. 67457488). The exposure-eligible source cohort was identified from the official mimiciv_derived.sepsis3 table. When antibiotics preceded culture, culture was required within 24 hours after antibiotic initiation; when culture preceded antibiotics, antibiotics were required within 72 hours after culture. The SOFA assessment window extended from 48 hours before to 24 hours after suspected infection. The exact mimic-code commit used to generate the retained sepsis3 table was not recorded; the dataset version, derived-table name and construction windows are therefore reported without assigning an unverifiable source-code version. The exposure-eligible source cohort defines endpoint lineage, whereas patients strictly alive beyond ICU admission +24 hours form the primary inferential risk set. MIMIC-IV uses reconstructed 28-day all-cause mortality from ICU admission; eICU uses a database-derived ICU 28-day endpoint, with patients discharged alive from the ICU before day 28 classified as alive at day 28; and the independent center uses traceable 28-day all-cause mortality. The independent-center cohort retained 492 of 615 reviewed records on the basis of complete locked SOFA-2 source fields and first-24-hour vasoactive-treatment evidence; its locked cohort-specific time origin was retained and it is used only as a directional stress test. Accordingly, eICU is a transportability cohort and the independent center is a high-risk stress-test cohort; neither is treated as endpoint-equivalent validation of MIMIC-IV absolute risk. SOFA, Sequential Organ Failure Assessment.

Supplementary Table S2. MIMIC-IV source-cohort baseline characteristics by reconstructed 28-day all-cause mortality

| **Characteristic** | **Overall** | **Survivors through ICU day 28** | **28-day all-cause deaths** | **Missing, n** | **P value** | **SMD** |
| --- | --- | --- | --- | --- | --- | --- |
| Age, years | 69.0 [59.0, 79.0] | 68.0 [58.0, 77.0] | 72.0 [61.0, 82.0] | 0 | <0.001 | 0.248 |
| Male sex | 7,212/11,997 (60.12%) | 5,667/9,205 (61.56%) | 1,545/2,792 (55.34%) | 0 | <0.001 | 0.127 |
| Charlson comorbidity index | 5.0 [3.0, 7.0] | 5.0 [3.0, 7.0] | 6.0 [4.0, 9.0] | 0 | <0.001 | 0.594 |
| Lactate, mmol/L | 2.7 [1.8, 4.3] | 2.5 [1.8, 3.8] | 3.5 [2.0, 6.8] | 1,183 | <0.001 | 0.551 |
| SOFA-1 total score | 7.0 [5.0, 10.0] | 7.0 [5.0, 9.0] | 10.0 [7.0, 13.0] | 0 | <0.001 | 0.881 |
| SOFA-2 total score | 8.0 [6.0, 10.0] | 7.0 [5.0, 9.0] | 10.0 [8.0, 13.0] | 0 | <0.001 | 0.887 |
| SOFA-1 cardiovascular score | 3.0 [1.0, 4.0] | 3.0 [1.0, 4.0] | 4.0 [3.0, 4.0] | 0 | <0.001 | 0.691 |
| SOFA-2 cardiovascular score | 2.0 [2.0, 3.0] | 2.0 [2.0, 3.0] | 3.0 [2.0, 4.0] | 0 | <0.001 | 0.669 |
| Five-agent NE-equivalent, μg/kg/min | 0.2 [0.1, 0.3] | 0.1 [0.1, 0.2] | 0.3 [0.1, 0.6] | 2 | <0.001 | 0.558 |
| Catecholamine-only NE-equivalent, μg/kg/min | 0.1 [0.1, 0.3] | 0.1 [0.1, 0.2] | 0.3 [0.1, 0.5] | 2 | <0.001 | 0.536 |
| Number of vasoactive agents | 1.0 [1.0, 2.0] | 1.0 [1.0, 2.0] | 1.0 [1.0, 2.0] | 0 | <0.001 | 0.463 |
| Norepinephrine use | 7,086/11,997 (59.06%) | 4,795/9,205 (52.09%) | 2,291/2,792 (82.06%) | 0 | <0.001 | 0.673 |
| Epinephrine use | 1,468/11,997 (12.24%) | 1,125/9,205 (12.22%) | 343/2,792 (12.29%) | 0 | 0.929 | 0.002 |
| Dopamine use | 759/11,997 (6.33%) | 475/9,205 (5.16%) | 284/2,792 (10.17%) | 0 | <0.001 | 0.189 |
| Phenylephrine use | 5,901/11,997 (49.19%) | 4,956/9,205 (53.84%) | 945/2,792 (33.85%) | 0 | <0.001 | 0.411 |
| Vasopressin use | 1,926/11,997 (16.05%) | 1,024/9,205 (11.12%) | 902/2,792 (32.31%) | 0 | <0.001 | 0.532 |
| Multiple vasoactive agents | 3,746/11,997 (31.22%) | 2,453/9,205 (26.65%) | 1,293/2,792 (46.31%) | 0 | <0.001 | 0.417 |

Note. This descriptive table includes the exposure-eligible source cohort of 11,997 patients, including 2,792 reconstructed 28-day all-cause deaths from ICU admission. Continuous variables are reported as median [interquartile range], and categorical variables as n/N (%); missing counts are shown separately and categorical denominators use nonmissing observations. P values comparing patients alive through ICU day 28 with those who died by day 28 were calculated using Wilcoxon rank-sum tests for continuous variables and Pearson chi-square tests for categorical variables. Standardized mean differences quantify between-group imbalance. NE, norepinephrine; SMD, standardized mean difference; SOFA, Sequential Organ Failure Assessment.

Supplementary Table S3. Completeness and analysis handling of variables in the MIMIC-IV exposure-eligible source cohort

| **Variable** | **Cohort N** | **Missing n** | **Missing %** | **Handling** |
| --- | --- | --- | --- | --- |
| age | 11,997 | 0 | 0.00% | No missing values in cohort |
| sex | 11,997 | 0 | 0.00% | No missing values in cohort |
| charlson | 11,997 | 0 | 0.00% | No missing values in cohort |
| lactate | 11,997 | 1,183 | 9.86% | Primary adjusted association models: 20-dataset MICE with predictive mean matching; complete‑case sensitivity reported in Supplementary Table S20 |
| sofa1_total | 11,997 | 0 | 0.00% | No missing values in cohort |
| sofa2_total | 11,997 | 0 | 0.00% | No missing values in cohort |
| sofa1_cv | 11,997 | 0 | 0.00% | No missing values in cohort |
| sofa2_cv | 11,997 | 0 | 0.00% | No missing values in cohort |
| gcs_min_24h_derived | 11,997 | 1 | 0.01% | Not entered as a separate covariate and not displayed as an observed GCS measure in main Table 1. The retained MIMIC-IV event-level SQL includes sedation exclusion, pre-sedation fallback and motor-score handling; this incomplete derived field was used only for assessability audit. |
| gcs_min_24h_assessable | 11,997 | 3,598 | 29.99% | Assessability audit only; 8,399 patients had an assessable value. This field did not replace the locked SOFA-2 neurologic component. |
| invasive_ventilation_24h | 11,997 | 0 | 0.00% | MIMIC‑IV Table 1 definition; invasive ventilation or tracheostomy overlapping ICU hours 0-24. Excludes noninvasive ventilation, high‑flow nasal cannula, and supplemental oxygen; 8,992 patients (75.0%). |
| any_oxygen_or_ventilation_24h | 11,997 | 0 | 0.00% | Broad respiratory‑support flag; includes invasive and noninvasive ventilation, high‑flow nasal cannula, and supplemental oxygen. Not labeled as mechanical ventilation and not used in Table 1; 11,231 patients (93.6%). |
| ne_equiv_full5 | 11,997 | 2 | 0.02% | Not imputed because this was the analysis exposure; models requiring the variable excluded the two missing records |
| ne_equiv_catonly | 11,997 | 2 | 0.02% | Not imputed because this was the analysis exposure; models requiring the variable excluded the two missing records |
| num_vasopressors | 11,997 | 0 | 0.00% | No missing values in cohort |
| norepi24 | 11,997 | 0 | 0.00% | No missing values in cohort |
| epi24 | 11,997 | 0 | 0.00% | No missing values in cohort |
| dopamine24 | 11,997 | 0 | 0.00% | No missing values in cohort |
| phenyl24 | 11,997 | 0 | 0.00% | No missing values in cohort |
| vasopressin24 | 11,997 | 0 | 0.00% | No missing values in cohort |
| multi_vaso24 | 11,997 | 0 | 0.00% | No missing values in cohort |
| death_28d_reconstructed (stored field: icu28) | 11,997 | 0 | 0.00% | Formal MIMIC-IV endpoint: reconstructed 28-day all-cause mortality from ICU admission, using admissions.deathtime with patients.dod fallback; 2,792/11,997 events. This stored analysis field is distinct from raw_icu_survival_time. |
| raw_icu_survival_time | 11,997 | 8,924 | 74.39% | Raw source field; missing in 8,924 records. It does not establish survival after live ICU discharge and is not treated as complete 28-day all‑cause follow‑up. |
| time_28d | 11,997 | 0 | 0.00% | Derived 28-day analysis time. In the primary 24-hour landmark analysis, patients alive beyond ICU admission +24 hours were followed through ICU day 28; live ICU or hospital discharge did not trigger early censoring. |

Note. The source cohort comprised 11,997 patients, including 2,792 reconstructed 28-day all-cause deaths from ICU admission. Missing counts and percentages use 11,997 as the denominator unless otherwise specified. Lactate was imputed by predictive mean matching in 20 datasets for the primary adjusted association models; outcomes, SOFA-1 and SOFA-2 scores, and vasoactive-drug exposures were retained as observed. Records lacking a finite five-agent or catecholamine-only norepinephrine-equivalent value were excluded only from analyses requiring that exposure. The assessability field for Glasgow Coma Scale did not replace the retained SOFA-2 neurologic component, and the raw ICU survival-time field was not treated as complete 28-day all-cause follow-up. GCS, Glasgow Coma Scale; ICU, intensive care unit; MICE, multivariate imputation by chained equations; NE, norepinephrine; SOFA, Sequential Organ Failure Assessment.

Supplementary Table S4. Vasopressor mapping and norepinephrine-equivalent conversion rules

**(A). Five-agent norepinephrine-equivalent conversion rules**

| **Drug** | **Main five‑agent NE‑equivalent conversion** | **Catecholamine‑only sensitivity handling** | **Included in strict five exposure definition** | **Distinct source itemids** | **Distinct source units** | **Source records** | **Included source records** | **Excluded source records** |
| --- | --- | --- | --- | --- | --- | --- | --- | --- |
| dopamine | dopamine rate / 100 | included in dose | Yes | 1 | μg/kg/min | 4,417 | 4,410 | 7 |
| epinephrine | epinephrine rate x 1 | included in dose | Yes | 2 | μg/kg/min; missing | 7,987 | 7,950 | 37 |
| norepinephrine | norepinephrine rate x 1 | included in dose | Yes | 1 | μg/kg/min | 77,531 | 77,503 | 28 |
| phenylephrine | phenylephrine rate / 10 | included in dose | Yes | 3 | μg/kg/min | 52,961 | 52,905 | 56 |
| vasopressin | (vasopressin rate in U/hour ÷ 60) × 2.5 | not dose‑converted; retained as separate vasoactive drug composition information | Yes | 1 | units/hour | 4,883 | 4,874 | 9 |

**(B). Inotrope mapping for SOFA-2 cardiovascular scoring**

| **Drug** | **Exact MIMIC-IV itemid** | **Normalized source field** | **First-24-hour positive-rate exposure** | **≥60-minute episode exposure** | **Included in SOFA-2 cardiovascular scoring** | **Included in five-agent NE-equivalent formula** | **Notes** |
| --- | --- | --- | --- | --- | --- | --- | --- |
| Dobutamine | 221653 | mimiciv_derived. vasoactive_agent. dobutamine | 300/11,997 (2.5%) | 265/11,997 (2.2%) | Yes | No | Implemented other vasopressor/inotrope field; qualifying exposure requires a merged episode of at least 1 hour. |
| Milrinone | 221986 | mimiciv_derived. vasoactive_agent. milrinone | 344/11,997 (2.9%) | 336/11,997 (2.8%) | Yes | No | Implemented other vasopressor/inotrope field; qualifying exposure requires a merged episode of at least 1 hour. |

Worked example. For a vasopressin infusion of 2.4 U/hour, the source rate is first converted to 0.04 U/min (2.4 ÷ 60); the corresponding NE-equivalent contribution is 0.10 μg/kg/min (0.04 × 2.5).

Note. The conversion rules are exposure-definition rules and are not altered by cohort restriction. itemid, database item identifier; NE, norepinephrine; SOFA, Sequential Organ Failure Assessment.

Supplementary Table S5. Catecholamine-only norepinephrine-equivalent sensitivity analysis in the MIMIC-IV 24-hour landmark cohort

| **Group** | **N** | **Events** | **Post-landmark mortality** | **Adjusted OR (95% CI)** | **P value** | **Model N/events** |
| --- | --- | --- | --- | --- | --- | --- |
| ≤0.1 | 3,801 | 440 | 440/3,801 (11.58%) | Reference | — | 11,890/2,686 |
| >0.1-0.3 | 5,163 | 1,014 | 1,014/5,163 (19.64%) | 1.55 (1.36–1.76) | <0.001 | 11,890/2,686 |
| >0.3-0.5 | 1,438 | 536 | 536/1,438 (37.27%) | 2.90 (2.47–3.41) | <0.001 | 11,890/2,686 |
| >0.5 | 1,488 | 696 | 696/1,488 (46.77%) | 3.48 (2.95–4.09) | <0.001 | 11,890/2,686 |

Note. The analysis included 11,890 patients who were alive beyond ICU admission +24 hours and had a finite catecholamine-only burden, including 2,686 post-landmark deaths by ICU day 28. Catecholamine-only norepinephrine-equivalent burden was calculated from norepinephrine, epinephrine, dopamine/100 and phenylephrine/10; vasopressin was not dose-converted in this sensitivity definition. Models adjusted for age, sex, Charlson comorbidity index, lactate and the sum of the five non-cardiovascular SOFA-2 domains. Lactate was imputed by predictive mean matching in 20 datasets; outcome and exposure variables were retained as observed. The ≤0.1 μg/kg/min category was the reference. Reported odds ratios are prognostic associations and do not estimate causal effects of drug selection or dose. CI, confidence interval; ICU, intensive care unit; OR, odds ratio; SOFA, Sequential Organ Failure Assessment.

Supplementary Table S6. Full multivariable logistic-regression models for post-landmark 28-day all-cause mortality in MIMIC-IV

| **Model** | **Term** | **Imputations** | **N/events per imputation** | **Adjusted OR** | **95% CI** | **P value** |
| --- | --- | --- | --- | --- | --- | --- |
| SOFA-2 total ≥12 vs <12 | Intercept | 20 | 11,892/2,687 | 0.03 | 0.02–0.04 | <0.001 |
| SOFA-2 total ≥12 vs <12 | SOFA-2 total ≥12 | 20 | 11,892/2,687 | 3.89 | 3.44–4.39 | <0.001 |
| SOFA-2 total ≥12 vs <12 | Age, per year | 20 | 11,892/2,687 | 1.01 | 1.00–1.01 | <0.001 |
| SOFA-2 total ≥12 vs <12 | Male sex | 20 | 11,892/2,687 | 0.77 | 0.70–0.85 | <0.001 |
| SOFA-2 total ≥12 vs <12 | Charlson index, per point | 20 | 11,892/2,687 | 1.23 | 1.20–1.25 | <0.001 |
| SOFA-2 total ≥12 vs <12 | Lactate, per mmol/L | 20 | 11,892/2,687 | 1.12 | 1.11–1.14 | <0.001 |
| SOFA-2 cardiovascular 3–4 vs 0–2 | Intercept | 20 | 11,892/2,687 | 0.01 | 0.01–0.02 | <0.001 |
| SOFA-2 cardiovascular 3–4 vs 0–2 | SOFA-2 cardiovascular 3–4 | 20 | 11,892/2,687 | 1.82 | 1.64–2.01 | <0.001 |
| SOFA-2 cardiovascular 3–4 vs 0–2 | Age, per year | 20 | 11,892/2,687 | 1.01 | 1.00–1.01 | <0.001 |
| SOFA-2 cardiovascular 3–4 vs 0–2 | Male sex | 20 | 11,892/2,687 | 0.74 | 0.68–0.82 | <0.001 |
| SOFA-2 cardiovascular 3–4 vs 0–2 | Charlson index, per point | 20 | 11,892/2,687 | 1.21 | 1.18–1.23 | <0.001 |
| SOFA-2 cardiovascular 3–4 vs 0–2 | Lactate, per mmol/L | 20 | 11,892/2,687 | 1.08 | 1.06–1.10 | <0.001 |
| SOFA-2 cardiovascular 3–4 vs 0–2 | Non-cardiovascular SOFA-2, per point | 20 | 11,892/2,687 | 1.22 | 1.20–1.24 | <0.001 |
| Five-agent NE-equivalent strata | Intercept | 20 | 11,890/2,686 | 0.01 | 0.01–0.01 | <0.001 |
| Five-agent NE-equivalent strata | >0.1–0.3 vs ≤0.1 | 20 | 11,890/2,686 | 1.57 | 1.38–1.79 | <0.001 |
| Five-agent NE-equivalent strata | >0.3–0.5 vs ≤0.1 | 20 | 11,890/2,686 | 2.68 | 2.28–3.15 | <0.001 |
| Five-agent NE-equivalent strata | >0.5 vs ≤0.1 | 20 | 11,890/2,686 | 3.60 | 3.07–4.23 | <0.001 |
| Five-agent NE-equivalent strata | Age, per year | 20 | 11,890/2,686 | 1.01 | 1.01–1.01 | <0.001 |
| Five-agent NE-equivalent strata | Male sex | 20 | 11,890/2,686 | 0.75 | 0.68–0.83 | <0.001 |
| Five-agent NE-equivalent strata | Charlson index, per point | 20 | 11,890/2,686 | 1.21 | 1.18–1.23 | <0.001 |
| Five-agent NE-equivalent strata | Lactate, per mmol/L | 20 | 11,890/2,686 | 1.06 | 1.04–1.08 | <0.001 |
| Five-agent NE-equivalent strata | Non-cardiovascular SOFA-2, per point | 20 | 11,890/2,686 | 1.20 | 1.18–1.22 | <0.001 |

Note. Patients were required to be alive beyond ICU admission +24 hours, and the outcome was death by ICU day 28. The SOFA-2 ≥12 and cardiovascular-score models included 11,892 patients and 2,687 events; models requiring finite five-agent norepinephrine-equivalent burden included 11,890 patients and 2,686 events. The exploratory SOFA-2 ≥12 model adjusted for age, sex, Charlson comorbidity index and lactate. Cardiovascular-score and norepinephrine-equivalent models additionally adjusted for the sum of the five non-cardiovascular SOFA-2 domains. Lactate was imputed by predictive mean matching in 20 datasets; outcomes, SOFA scores and exposures were retained as observed. Odds ratios and 95% confidence intervals were pooled across imputations. These models estimate prognostic associations and do not identify causal treatment effects. CI, confidence interval; ICU, intensive care unit; NE, norepinephrine; OR, odds ratio; SOFA, Sequential Organ Failure Assessment.

Supplementary Table S7. Discrimination and overall prediction error for post-landmark 28-day all-cause mortality in MIMIC-IV

| **Model** | **N** | **Events** | **AUROC (95% CI)** | **Brier score** | **Comparator** | **DeLong P** |
| --- | --- | --- | --- | --- | --- | --- |
| SOFA-1 total | 11,892 | 2,687 | 0.732 (0.721–0.742) | 0.152 | — | — |
| SOFA-2 total | 11,892 | 2,687 | 0.731 (0.720–0.742) | 0.151 | SOFA-1 total | 0.908 |
| SOFA-1 cardiovascular | 11,892 | 2,687 | 0.676 (0.666–0.686) | 0.162 | — | — |
| SOFA-2 cardiovascular | 11,892 | 2,687 | 0.663 (0.652–0.674) | 0.162 | SOFA-1 cardiovascular | 0.006 |
| M0: non-cardiovascular SOFA-2 | 11,890 | 2,686 | 0.708 (0.696–0.719) | 0.155 | — | — |
| M3: M0 + cardiovascular score + continuous burden | 11,890 | 2,686 | 0.743 (0.732–0.754) | 0.150 | M0 | — |
| M4: M0 + five vasoactive-drug indicators | 11,890 | 2,686 | 0.763 (0.753–0.773) | 0.147 | M0 | — |
| SOFA-2 total + five drug indicators | 11,890 | 2,686 | 0.768 (0.758–0.778) | 0.146 | SOFA-2 total | <0.001 |

Note. SOFA-1 and SOFA-2 score comparisons used the 11,892 patients who were alive beyond ICU admission +24 hours, including 2,687 deaths by ICU day 28. Prediction models requiring a finite continuous burden used the common prediction subset of 11,890 patients and 2,686 events. M0 contains the five non-cardiovascular SOFA-2 domains; M3 adds the SOFA-2 cardiovascular score and continuous five-agent norepinephrine-equivalent burden; M4 adds five non-mutually-exclusive first-24-hour drug indicators. AUROC 95% confidence intervals and paired DeLong P values were calculated on identical patient subsets. Brier score denotes mean squared prediction error. The composition model contains SOFA-2 total and the five drug indicators. AUROC, area under the receiver operating characteristic curve; CI, confidence interval; ICU, intensive care unit; NE, norepinephrine; SOFA, Sequential Organ Failure Assessment.

Supplementary Table S7 (continued). Bootstrap optimism correction for post-landmark 28-day mortality models

| **Model** | **N** | **Events** | **Bootstrap resamples** | **Apparent AUROC** | **Corrected AUROC** | **Apparent Brier** | **Corrected Brier** |
| --- | --- | --- | --- | --- | --- | --- | --- |
| SOFA-2 total: full landmark cohort | 11,892 | 2,687 | 1,000 | 0.731 | 0.731 | 0.151 | 0.152 |
| SOFA-2 total: common prediction subset | 11,890 | 2,686 | 1,000 | 0.731 | 0.731 | 0.151 | 0.151 |
| SOFA-2 total + five drug indicators | 11,890 | 2,686 | 1,000 | 0.768 | 0.767 | 0.146 | 0.146 |

Note. The full 24-hour landmark cohort comprised 11,892 patients and 2,687 post-landmark deaths; the full-cohort SOFA-2 score model was internally validated with 1,000 bootstrap resamples using seed 20260824. The common prediction subset comprised 11,890 patients and 2,686 deaths and was used for the subset SOFA-2 model and the model containing SOFA-2 total plus five first-24-hour drug indicators. Apparent and optimism-corrected AUROC and Brier scores are shown separately for each analysis set. These are internal development-sample estimates rather than external validation. AUROC, area under the receiver operating characteristic curve; ICU, intensive care unit; SOFA, Sequential Organ Failure Assessment.

Supplementary Table S8. External-cohort endpoints and adjudication roles

| **Cohort** | **Primary endpoint** | **N** | **Events** | **Event rate** | **Cohort construction** |
| --- | --- | --- | --- | --- | --- |
| eICU transportability cohort | Database-derived ICU 28-day endpoint | 1,528 | 441 | 28.86% | Structured sepsis/septic shock diagnosis within ICU admission ±24 h plus complete first‑day SOFA ≥2 The strict cohort required ICU stay and survival beyond 24 hours; fixed strata were assessed at the 24-hour index. |
| Independent-center stress-test cohort | Traceable 28-day all‑cause mortality | 492 | 298 | 60.57% | Clinical diagnosis record plus case-level final clinician adjudication of infection and SOFA increase ≥2. Of 615 reviewed records, 492 with complete locked SOFA-2 source fields and first-24-hour vasoactive-treatment evidence were retained. The retained cohort had 298/492 deaths (60.57%); the full source cohort had 322/615 deaths (52.36%); the 123 excluded records had 24 deaths (19.51%), demonstrating selection enrichment. All exclusions lacked complete source fields, and 107 also lacked early vasoactive-treatment evidence. Eligible ICU admissions were recorded from 1 January 2023 to 1 April 2026; 28-day all-cause mortality was ascertainable for all included patients. All included patients remained in the ICU and were alive beyond 24 hours; fixed strata were assessed at the 24-hour index; the cohort is used only as a directional stress test. |

Note. eICU is a transportability cohort with a database-derived ICU endpoint; the independent center is a high-risk stress-test cohort with traceable 28-day all-cause mortality. ICU, intensive care unit.

Supplementary Table S9. SOFA-2 score 0–4 definitions by organ domain, retained evidence and reproducibility status

| **Domain / rule** | **Documented score specification** | **Retained source evidence** | **Reproducibility status** |
| --- | --- | --- | --- |
| Respiratory | Score 0: P/F >300 mm Hg (>40 kPa); if P/F is unavailable, S/F >300.  Score 1: P/F >225 to ≤300 mm Hg, or S/F >250 to ≤300.  Score 2: P/F >150 to ≤225 mm Hg, or S/F >200 to ≤250.  Score 3: P/F >75 to ≤150 mm Hg, or S/F >120 to ≤200, with advanced ventilatory support.  Score 4: P/F ≤75 mm Hg (≤10 kPa), or S/F ≤120, with advanced ventilatory support; or ECMO.  Rule: S/F is used only when P/F is unavailable (and SpO₂ <98%). Advanced support comprises HFNO, CPAP/NIV, invasive mechanical ventilation or home ventilation. Without advanced support, the maximum is 2 unless support is unavailable or precluded by a ceiling of treatment. | Locked respiratory component in all cohorts; executable MIMIC-IV respiratory, oxygenation and support mappings with QC outputs. | MIMIC-IV locally reconstructable; the current eICU 1,528-patient and independent-center raw pathways were not fully retained. |
| Hemostasis (coagulation) | Score 0: Platelets >150 ×10³/µL. Score 1: Platelets >100 to ≤150 ×10³/µL. Score 2: Platelets >80 to ≤100 ×10³/µL. Score 3: Platelets >50 to ≤80 ×10³/µL. Score 4: Platelets ≤50 ×10³/µL. Rule: Use the lowest eligible platelet count in the first 24 hours; thresholds are evaluated in descending severity order. | Locked hemostasis component in all cohorts; executable MIMIC-IV platelet mapping and boundary-value QC retained. | MIMIC-IV scoring SQL verified against the JAMA 2025 definition at 50, 80, 100 and 150 ×10³/µL; outside MIMIC-IV, the final component was verified but raw-event lineage was incomplete. |
| Liver | Score 0: Total bilirubin ≤1.20 mg/dL (≤20.6 µmol/L).  Score 1: Total bilirubin >1.20 to ≤3.0 mg/dL (>20.6 to ≤51.3 µmol/L).  Score 2: Total bilirubin >3.0 to ≤6.0 mg/dL (>51.3 to ≤102.6 µmol/L).  Score 3: Total bilirubin >6.0 to ≤12.0 mg/dL (>102.6 to ≤205 µmol/L).  Score 4: Total bilirubin >12.0 mg/dL (>205 µmol/L).  Rule: Use the highest eligible total bilirubin in the first 24 hours. | Locked liver component in all cohorts; executable MIMIC-IV bilirubin mapping with QC outputs. | MIMIC-IV locally reconstructable; outside MIMIC-IV, the final component was verified but raw-event lineage was incomplete. |
| Cardiovascular | Score 0: MAP ≥70 mm Hg with no qualifying vasopressor/inotrope or mechanical circulatory support.  Score 1: MAP <70 mm Hg with no qualifying vasopressor/inotrope or mechanical circulatory support.  Score 2: Norepinephrine + epinephrine >0 to ≤0.2 µg/kg/min without another agent; any qualifying other vasopressor/inotrope; or dopamine alone ≤20 µg/kg/min.  Score 3: Norepinephrine + epinephrine >0.2 to ≤0.4 µg/kg/min without another agent; >0 to ≤0.2 µg/kg/min with another agent; or dopamine alone >20 to ≤40 µg/kg/min.  Score 4: Norepinephrine + epinephrine >0.4 µg/kg/min; >0.2 to ≤0.4 µg/kg/min with another agent; dopamine alone >40 µg/kg/min; or qualifying mechanical circulatory support.  Rule: Mirrors Table S16. Qualifying infusions last ≥1 hour; another agent denotes dopamine, dobutamine, vasopressin, phenylephrine or milrinone. Mechanical support includes VA/VAV-ECMO, IABP, LVAD and microaxial-flow pumps. The maximum qualifying state is assigned. | MIMIC-IV itemids, rates, duration logic, MAP and device mappings; independent-center exposure-duration and VA-ECMO audits; locked eICU component. | MIMIC-IV locally reconstructable; the independent center was partially reconstructable, but 191 exposures lacked confirmed ≥1-hour duration; the current eICU raw pathway was not retained. |
| Brain (neurologic) | Score 0: GCS 15; if the total GCS is unassessable, motor 6 (obeys commands).  Score 1: GCS 13–14; motor 5 (localizes pain); or need for drugs to treat delirium.  Score 2: GCS 9–12; or motor 4 (withdraws from pain).  Score 3: GCS 6–8; or motor 3 (flexion to pain).  Score 4: GCS 3–5; or motor ≤2 (extension/no response; generalized myoclonus is also score 4).  Rule: During documented sedation, use the most recent assessable pre-sedation GCS within 24 hours; if unavailable, the retained MIMIC implementation assigns 0 unless delirium treatment imposes a minimum score of 1. | MIMIC-IV event-level GCS, sedation, motor-fallback and pre-sedation logic; independent-center GCS fields; locked eICU component. | MIMIC-IV locally reconstructable. These fallback rules are not asserted for the independent center or current eICU cohort because their event-level sedation/unassessable pathways were not retained; independent-center GCS-to-domain agreement was 492/492. |
| Kidney (renal) | Score 0: Creatinine ≤1.20 mg/dL (≤110 µmol/L), without a higher urine-output or RRT criterion.  Score 1: Creatinine >1.20 to ≤2.0 mg/dL (>110 to ≤170 µmol/L), or urine output <0.5 mL/kg/h for 6 to <12 hours.  Score 2: Creatinine >2.0 to ≤3.50 mg/dL (>170 to ≤300 µmol/L), or urine output <0.5 mL/kg/h for ≥12 hours.  Score 3: Creatinine >3.50 mg/dL (>300 µmol/L), urine output <0.3 mL/kg/h for ≥24 hours, or anuria for ≥12 hours.  Score 4: Receiving RRT for a renal indication (including chronic RRT), or meeting RRT criteria when treatment is unavailable, delayed or precluded: creatinine >1.2 mg/dL or urine output <0.3 mL/kg/h for >6 hours, plus potassium ≥6.0 mmol/L or metabolic acidosis with pH ≤7.20 and bicarbonate ≤12 mmol/L.  Rule: RRT exclusively for nonrenal indications is excluded; intermittent RRT remains score 4 on off-days until RRT is terminated. The retained MIMIC pathway prioritizes RRT, then virtual-RRT criteria, creatinine and observed weight-normalized urine-output windows; an unobserved urine window is not anuria. | MIMIC-IV RRT, virtual-RRT, creatinine and weight-normalized urine-output logic; independent-center RRT/CRRT fields; locked eICU component. | MIMIC-IV locally reconstructable. All 178 independent-center RRT/CRRT patients had kidney score 4, but the independent-center RRT/creatinine/urine priority and urine-duration pathway were not retained; the current eICU raw pathway was not retained. |
| Dobutamine / milrinone | Qualifying infusions are other vasopressor/inotropic agents for cardiovascular scoring. | MIMIC itemids 221653 and 221986 and duration audit retained. | Included in SOFA-2 cardiovascular scoring when qualifying; excluded from the five-agent norepinephrine-equivalent formula. |
| Mechanical circulatory support | Qualifying VA-ECMO, IABP, LVAD or microaxial-flow support gives cardiovascular score 4. | MIMIC-IV support mappings retained; independent-center registry cross-check identified 12 VA-ECMO cases; final cardiovascular component available in all cohorts. | MIMIC-IV locally reconstructable; independent-center VA-ECMO was partially verified, while IABP/LVAD/microaxial fields and the current eICU raw mapping were not retained. |
| Total score | Arithmetic sum of the six retained domain scores. | Six component fields and total field in MIMIC-IV, eICU and the independent center. | Patient-level arithmetic agreement: 11,997/11,997; 1,528/1,528; 492/492. Arithmetic agreement is not raw-event reconstruction. |

Note. The six organ-domain rows reproduce the published SOFA-2 score 0–4 definitions in a common format; the cardiovascular row mirrors Table S16 while the retained-evidence and reproducibility columns report this study's implementation lineage. MIMIC-IV retained executable local SQL and quality-control outputs for all six domains, although the exact mimic-code commit used to build the derivative table was not recorded. In the retained MIMIC neurologic implementation, unassessable or missing total GCS can use the motor component, observations during documented sedation are excluded, and the latest assessable pre-sedation GCS within 24 hours is used as fallback; the renal implementation prioritizes first-24-hour RRT, then virtual-RRT criteria, creatinine and observed weight-normalized urine-output windows, without treating a completely unobserved urine window as anuria. In the independent center, retained-field checks verified GCS-to-neurologic score agreement in 492/492 patients and RRT/CRRT-to-kidney score 4 in 178/178; however, sedation/unassessable timing, urine-duration data and duration confirmation for 191 vasoactive exposures were unavailable, so the detailed MIMIC fallback and priority rules are not asserted for that cohort. An event-level eICU reconstruction existed for a separate 633-patient unit-death analysis and was not treated as evidence for the current 1,528-patient transportability cohort. Arithmetic agreement between the six retained domain scores and SOFA-2 total was verified in 11,997/11,997 MIMIC-IV patients, 1,528/1,528 eICU patients and 492/492 independent-center patients. These checks support arithmetic and prognostic validation of the locked SOFA-2 fields but do not establish implementation equivalence across cohorts. ECMO, extracorporeal membrane oxygenation; GCS, Glasgow Coma Scale; IABP, intra-aortic balloon pump; LVAD, left ventricular assist device; MAP, mean arterial pressure; P/F, ratio of arterial oxygen partial pressure to inspired oxygen fraction; RRT, renal replacement therapy; S/F, ratio of peripheral oxygen saturation to inspired oxygen fraction; SOFA, Sequential Organ Failure Assessment.

Supplementary Table S10. Observed and standardized 28-day mortality across SOFA-2 and vasoactive-support strata

| **Exposure** | **Group** | **N** | **Events** | **Observed mortality; standardized risk; adjusted RD vs reference (95% CI)** |
| --- | --- | --- | --- | --- |
| SOFA-2 total stratum | <12 | 10,013 | 1,711 | Observed 1,711/10,013 (17.09%); standardized 18.31%; RD reference |
| SOFA-2 total stratum | ≥12 | 1,879 | 976 | Observed 976/1,879 (51.94%); standardized 43.04%; adjusted RD 24.73 pp (22.22–27.23) |
| SOFA-2 cardiovascular score stratum | 0-2 | 7,389 | 1,125 | Observed 1,125/7,389 (15.23%); standardized 18.77%; RD reference |
| SOFA-2 cardiovascular score stratum | 3-4 | 4,503 | 1,562 | Observed 1,562/4,503 (34.69%); standardized 27.59%; adjusted RD 8.81 pp (7.27–10.35) |
| Five-agent NE-equivalent, μg/kg/min | ≤0.1 | 3,753 | 424 | Observed 424/3,753 (11.30%); standardized 15.05% (13.84–16.26); RD reference |
| Five-agent NE-equivalent, μg/kg/min | >0.1-0.3 | 5,023 | 974 | Observed 974/5,023 (19.39%); standardized 20.75% (19.69–21.81); adjusted RD 5.70 pp (4.11–7.28) |
| Five-agent NE-equivalent, μg/kg/min | >0.3-0.5 | 1,449 | 498 | Observed 498/1,449 (34.37%); standardized 29.17% (27.09–31.25); adjusted RD 14.12 pp (11.68–16.56) |
| Five-agent NE-equivalent, μg/kg/min | >0.5 | 1,665 | 790 | Observed 790/1,665 (47.45%); standardized 34.51% (32.32–36.69); adjusted RD 19.46 pp (16.86–22.05) |
| Catecholamine-only NE-equivalent, μg/kg/min | ≤0.1 | 3,801 | 440 | Observed 440/3,801 (11.58%); standardized RD not estimated for this sensitivity exposure |
| Catecholamine-only NE-equivalent, μg/kg/min | >0.1-0.3 | 5,163 | 1,014 | Observed 1,014/5,163 (19.64%); standardized RD not estimated for this sensitivity exposure |
| Catecholamine-only NE-equivalent, μg/kg/min | >0.3-0.5 | 1,438 | 536 | Observed 536/1,438 (37.27%); standardized RD not estimated for this sensitivity exposure |
| Catecholamine-only NE-equivalent, μg/kg/min | >0.5 | 1,488 | 696 | Observed 696/1,488 (46.77%); standardized RD not estimated for this sensitivity exposure |

Note. The primary cohort comprised 11,892 patients who were alive beyond ICU admission +24 hours, including 2,687 deaths by ICU day 28. The SOFA-2 ≥12 rule was derived exploratorily in this cohort. Cardiovascular scores were grouped as 0–2 and 3–4. Five-agent norepinephrine-equivalent peak burden was calculated as norepinephrine + epinephrine + dopamine/100 + phenylephrine/10 + vasopressin (U/min) ×2.5 and grouped into four study-defined strata (≤0.1, >0.1–0.3, >0.3–0.5 and >0.5 μg/kg/min). Two patients lacked a finite continuous burden, leaving 11,890 patients and 2,686 events for burden analyses. The catecholamine-only sensitivity definition omitted vasopressin dose conversion. These strata describe prognosis and are not treatment thresholds. ICU, intensive care unit; SOFA, Sequential Organ Failure Assessment.

Supplementary Table S11. SOFA-2 total-score distribution and post-landmark 28-day all-cause mortality in MIMIC-IV

| **SOFA-2 total score** | **N** | **Events** | **Exploratory stratum** | **Post-landmark 28-day all-cause mortality** |
| --- | --- | --- | --- | --- |
| 1 | 9 | 0 | <12 | 0/9 (0.00%) |
| 2 | 153 | 8 | <12 | 8/153 (5.23%) |
| 3 | 424 | 23 | <12 | 23/424 (5.42%) |
| 4 | 796 | 62 | <12 | 62/796 (7.79%) |
| 5 | 1,268 | 104 | <12 | 104/1,268 (8.20%) |
| 6 | 1,601 | 197 | <12 | 197/1,601 (12.30%) |
| 7 | 1,666 | 277 | <12 | 277/1,666 (16.63%) |
| 8 | 1,423 | 275 | <12 | 275/1,423 (19.33%) |
| 9 | 1,099 | 264 | <12 | 264/1,099 (24.02%) |
| 10 | 895 | 264 | <12 | 264/895 (29.50%) |
| 11 | 679 | 237 | <12 | 237/679 (34.90%) |
| 12 | 494 | 205 | ≥12 | 205/494 (41.50%) |
| 13 | 402 | 200 | ≥12 | 200/402 (49.75%) |
| 14 | 282 | 130 | ≥12 | 130/282 (46.10%) |
| 15 | 224 | 123 | ≥12 | 123/224 (54.91%) |
| 16 | 159 | 93 | ≥12 | 93/159 (58.49%) |
| 17 | 104 | 66 | ≥12 | 66/104 (63.46%) |
| 18 | 74 | 50 | ≥12 | 50/74 (67.57%) |
| 19 | 51 | 39 | ≥12 | 39/51 (76.47%) |
| 20 | 44 | 30 | ≥12 | 30/44 (68.18%) |
| 21 | 26 | 23 | ≥12 | 23/26 (88.46%) |
| 22 | 11 | 9 | ≥12 | 9/11 (81.82%) |
| 23 | 6 | 6 | ≥12 | 6/6 (100.00%) |
| 24 | 2 | 2 | ≥12 | 2/2 (100.00%) |

Note. The table includes 11,892 patients who were alive beyond ICU admission +24 hours, including 2,687 deaths by ICU day 28. N and events are reported at each observed integer SOFA-2 total score. Scores 1–11 form the exploratory <12 stratum, and scores 12–24 form the exploratory ≥12 stratum. This data-derived grouping is intended for prognostic stratification and is not a stand-alone treatment-escalation threshold. ICU, intensive care unit; SOFA, Sequential Organ Failure Assessment.

Supplementary Table S12. Model definitions, analysis denominators and procedures for post-landmark 28-day mortality

| **Model** | **Role** | **Predictors** | **Candidate N** | **Candidate events** | **Analysis N** | **Analysis events** | **Excluded** | **Procedure** |
| --- | --- | --- | --- | --- | --- | --- | --- | --- |
| SOFA-1 total | Discrimination | SOFA-1 total | 11,892 | 2,687 | 11,892 | 2,687 | 0 | Paired DeLong |
| SOFA-2 total | Discrimination | SOFA-2 total | 11,892 | 2,687 | 11,892 | 2,687 | 0 | Paired DeLong; bootstrap |
| SOFA-1 cardiovascular | Discrimination | SOFA-1 cardiovascular | 11,892 | 2,687 | 11,892 | 2,687 | 0 | Paired DeLong |
| SOFA-2 cardiovascular | Discrimination | SOFA-2 cardiovascular | 11,892 | 2,687 | 11,892 | 2,687 | 0 | Paired DeLong |
| SOFA-2 ≥12 | Association | Threshold + covariates | 11,892 | 2,687 | 11,892 | 2,687 | 0 | 20-dataset MI |
| Cardiovascular 3–4 | Association | CV stratum + covariates + non-cardiovascular SOFA-2 | 11,892 | 2,687 | 11,892 | 2,687 | 0 | 20-dataset MI |
| NE burden strata | Association | Burden strata + covariates + non-cardiovascular SOFA-2 | 11,892 | 2,687 | 11,890 | 2,686 | 2 | 20-dataset MI |
| M0 | Prediction | Five non-cardiovascular domains | 11,892 | 2,687 | 11,890 | 2,686 | 2 | Complete prediction subset |
| M3 | Prediction | M0 + CV + continuous burden | 11,892 | 2,687 | 11,890 | 2,686 | 2 | Complete prediction subset |
| M4 | Prediction | M0 + five drug indicators | 11,892 | 2,687 | 11,890 | 2,686 | 2 | Complete prediction subset |
| Continuous five-agent NE burden only | Prediction | ne_equiv_full5 | 11,892 | 2,687 | 11,890 | 2,686 | 2 | Complete prediction subset; Figure 5/Supplementary Figure S5 DCA |
| SOFA-2 total + continuous five-agent NE burden | Prediction | sofa2_total + ne_equiv_full5 | 11,892 | 2,687 | 11,890 | 2,686 | 2 | Complete prediction subset; Figure 5/Supplementary Figure S5 DCA |
| Composition | Prediction | SOFA-2 + five drug indicators | 11,892 | 2,687 | 11,890 | 2,686 | 2 | 1,000 bootstrap resamples |

Note. Candidate denominators refer to the 11,892 MIMIC-IV patients who were alive beyond ICU admission +24 hours, including 2,687 deaths by ICU day 28. Score-only discrimination and the SOFA-2 ≥12 and cardiovascular-score association models used all 11,892 patients. Two patients lacked a finite five-agent norepinephrine-equivalent burden, leaving 11,890 patients and 2,686 events for burden and common-subset prediction models. M0 contains the five non-cardiovascular SOFA-2 domains; M3 adds the cardiovascular score and continuous burden; M4 adds five non-mutually-exclusive first-24-hour drug indicators; the composition model contains SOFA-2 total and the five drug indicators. Association estimates were pooled across 20 imputed datasets when indicated, paired score comparisons used DeLong tests, and the stated score/composition models underwent 1,000-resample bootstrap internal validation. CV, cardiovascular; ICU, intensive care unit; MI, multiple imputation; NE, norepinephrine; SOFA, Sequential Organ Failure Assessment.

Supplementary Table S13. Fixed SOFA-2 ≥12 strata in the external transportability and stress-test cohorts

**(A). Fixed SOFA-2 ≥12 strata in external cohorts**

| **Cohort** | **Exposure** | **Group** | **N** | **Events** | **Cohort-specific mortality endpoint** |
| --- | --- | --- | --- | --- | --- |
| eICU transportability cohort | SOFA-2 total stratum | <12 | 1,059 | 206 | Database-derived ICU 28-day endpoint |
| eICU transportability cohort | SOFA-2 total stratum | ≥12 | 469 | 235 | Database-derived ICU 28-day endpoint |
| eICU transportability cohort | SOFA-2 cardiovascular score stratum | 0-2 | 1,027 | 237 | Database-derived ICU 28-day endpoint |
| eICU transportability cohort | SOFA-2 cardiovascular score stratum | 3-4 | 501 | 204 | Database-derived ICU 28-day endpoint |
| Independent-center stress-test cohort | SOFA-2 total stratum | <12 | 331 | 180 | 180/331 (54.38%) |
| Independent-center stress-test cohort | SOFA-2 total stratum | ≥12 | 161 | 118 | 118/161 (73.29%) |
| Independent-center stress-test cohort | SOFA-2 cardiovascular score stratum | 0-2 | 354 | 190 | 190/354 (53.67%) |
| Independent-center stress-test cohort | SOFA-2 cardiovascular score stratum | 3-4 | 138 | 108 | 108/138 (78.26%) |

Note. The MIMIC-IV landmark-derived ≥12 rule was applied without re-estimation.

Supplementary Table S13 (continued).
(B). Landmark derivation and threshold stability in MIMIC-IV

| **Assessment item** | **Result** |
| --- | --- |
| Analysis type | Primary 24-hour landmark analysis |
| Source cohort | 11,997 patients; 2,792 28-day all-cause deaths |
| Deaths at or before landmark | 105 |
| Primary landmark cohort | 11,892 patients; 2,687 post-landmark deaths |
| Time zero | ICU admission +24 hours |
| Follow-up horizon | Through ICU day 28; maximum 27 days after landmark |
| Selected cutpoint | SOFA-2 ≥12 |
| Bootstrap selection | ≥12: 65.4%; ≥11: 28.7% |
| Observed mortality | <12: 17.09%; ≥12: 51.94% |
| Adjusted association | OR 3.89 (95% CI 3.44–4.39) |
| Adjusted absolute risk difference | 24.73 percentage points (95% CI 22.22–27.23) |
| Interpretation | Exploratory risk-stratification rule; not a treatment threshold |

Note. The source cohort contained 11,997 patients and 2,792 deaths. After excluding 105 deaths at or before 24 hours, the primary cohort contained 11,892 patients and 2,687 post-landmark deaths. The ≥12 cutpoint was selected in 65.4% of 1,000 bootstrap samples; ≥11 was selected in 28.7%. SOFA, Sequential Organ Failure Assessment.

Supplementary Table S14. Adjacent-cutpoint sensitivity and exploratory threshold performance for post-landmark 28-day mortality

| **Analysis** | **Exposure** | **Contrast** | **N** | **Events** | **Estimate (95% CI)** | **P value** |
| --- | --- | --- | --- | --- | --- | --- |
| Primary MI | SOFA-2 ≥12 | ≥12 vs <12 | 11,892 | 2,687 | OR 3.89 (3.44–4.39) | <0.001 |
| Adjacent cutpoint | SOFA-2 ≥11 | ≥11 vs <11 | 11,892 | 2,687 | Observed OR 4.81 | <0.001 |
| Observed risk | SOFA-2 ≥12 | 51.94% vs 17.09% | 11,892 | 2,687 | RD 34.85 pp (32.47–37.22) | <0.001 |
| Standardized risk | SOFA-2 ≥12 | 43.04% vs 18.31% | 11,892 | 2,687 | Adjusted RD 24.73 pp (22.22–27.23) | <0.001 |
| Bootstrap | SOFA-2 ≥12 | Selection frequency | 1,000 resamples | — | 65.4% | — |
| Bootstrap | SOFA-2 ≥11 | Selection frequency | 1,000 resamples | — | 28.7% | — |

Note. Analyses included 11,892 MIMIC-IV patients who were alive beyond ICU admission +24 hours, including 2,687 deaths by ICU day 28. The primary SOFA-2 ≥12 model adjusted for age, sex, Charlson comorbidity index and lactate; lactate was imputed by predictive mean matching in 20 datasets, whereas the outcome and SOFA-2 threshold were retained as observed. Standardized risks were obtained by averaging model-based predicted probabilities over the cohort after setting every patient to each threshold level in turn; adjusted risk differences contrast these standardized risks. Within-imputation variances and 95% confidence intervals were derived by the delta method from model gradients, and estimates and variances were combined across 20 imputed datasets using Rubin's rules. Observed risks and adjusted odds ratios are reported as shown. Selection frequency was assessed across 1,000 bootstrap samples. SOFA-2 ≥12 is the current data-derived exploratory landmark rule; SOFA-2 ≥11 is shown only as an adjacent-cutpoint sensitivity analysis. Neither cutpoint is a stand-alone treatment threshold. CI, confidence interval; ICU, intensive care unit; OR, odds ratio; RD, risk difference; SOFA, Sequential Organ Failure Assessment.

Supplementary Table S14 (continued). Diagnostic characteristics and total-score discrimination at the 24-hour landmark

| **Analysis** | **Metric/model** | **N** | **Events** | **Estimate** | **Interpretation** |
| --- | --- | --- | --- | --- | --- |
| Threshold ≥12 | Sensitivity / specificity | 11,892 | 2,687 | 36.32% / 90.19% | High specificity; limited sensitivity |
| Threshold ≥12 | PPV / NPV | 11,892 | 2,687 | 51.94% / 82.91% | Population-dependent predictive values |
| Total-score discrimination | SOFA-1 | 11,892 | 2,687 | AUROC 0.732 | Reference |
| Total-score discrimination | SOFA-2 | 11,892 | 2,687 | AUROC 0.731; P=0.908 | No overall advantage |
| Threshold ≥12 | High-risk proportion | 11,892 | 2,687 | 1,879/11,892 (15.80%) | 15.80% of the landmark cohort |
| Threshold ≥12 | LR+ / LR− | 11,892 | 2,687 | 3.70 (3.42–4.01) / 0.706 (0.686–0.727) | Likelihood ratios for the exploratory rule |

Note. Sensitivity, specificity, positive predictive value, negative predictive value, positive and negative likelihood ratios, and the high-risk proportion for SOFA-2 ≥12 were calculated in the same 11,892-patient MIMIC-IV landmark cohort with 2,687 post-landmark deaths by ICU day 28. SOFA-1 and SOFA-2 total-score AUROCs were calculated on the same patients and compared using a paired DeLong test. Predictive values depend on the event prevalence in this cohort. These metrics characterize prognosis and do not define treatment response or a treatment-escalation threshold. AUROC, area under the receiver operating characteristic curve; ICU, intensive care unit; LR, likelihood ratio; NPV, negative predictive value; PPV, positive predictive value; SOFA, Sequential Organ Failure Assessment.

Supplementary Table S15. Infection-source distribution and reproducibility assessment

**(A). Infection-source distribution across the study cohorts**

| **Infection source** | **MIMIC-IV exposure-eligible source cohort** | **eICU transportability cohort** | **Independent-center stress-test cohort** |
| --- | --- | --- | --- |
| Pulmonary | 4,227/11,997 (35.2%) | 535/1,528 (35.0%) | 331/492 (67.3%) |
| Urinary | 2,203/11,997 (18.4%) | 278/1,528 (18.2%) | 101/492 (20.5%) |
| Intra‑abdominal/hepatobiliary | 1,577/11,997 (13.1%) | 161/1,528 (10.5%) | 97/492 (19.7%) |
| Bloodstream/endovascular | 578/11,997 (4.8%) | 128/1,528 (8.4%) | NA (not separately captured) |
| Skin/soft tissue/bone/joint | 715/11,997 (6.0%) | 94/1,528 (6.2%) | NA (not separately captured) |
| Central nervous system | 111/11,997 (0.9%) | 16/1,528 (1.0%) | 17/492 (3.5%) |
| Other or unknown (no named source indicator) | 5,230/11,997 (43.6%) | 604/1,528 (39.5%) | 94/492 (19.1%) |

**(B). Availability of infection-source covariates for the current landmark reanalysis**

| **Information** | **Source-cohort availability** | **Primary-model use** | **Missing-data handling** | **Sensitivity analysis** | **Interpretation** | **Location** |
| --- | --- | --- | --- | --- | --- | --- |
| Six infection-source indicators | Available for descriptive reporting | Not included | No imputation | Not estimable from retained landmark file | Baseline case-mix description only | Main Table 1 (landmark cohort); Table S15A (source cohort) |
| Pulmonary source | Available | Not included | Observed values | Not performed | Non-mutually exclusive descriptive category | Main Table 1 (landmark cohort); Table S15A (source cohort) |
| Urinary source | Available | Not included | Observed values | Not performed | Non-mutually exclusive descriptive category | Main Table 1 (landmark cohort); Table S15A (source cohort) |
| Other source categories | Availability differs by cohort | Not included | Unavailable categories shown as NA | Not performed | NA does not denote absence | Main Table 1 (landmark cohort); Table S15A (source cohort) |
| Inference boundary | Source fields were not retained in the final landmark modeling file | No adjusted claim | Not applicable | Not claimed | Infection source is not presented as a confounder-adjusted result | Methods; limitations |

Supplementary Table S15 (continued).
Notes. Panel A preserves descriptive source-cohort distributions; for MIMIC-IV its denominator is the exposure-eligible source cohort (N=11,997), whereas main Table 1 reports the linked 24-h landmark primary cohort (N=11,892). Panel B reports source-cohort field availability and whether those indicators were retained in the current landmark modeling file; it does not change the Panel A denominator. Infection-source indicators were not present in the locked current landmark analysis file, so an infection-source-adjusted numerical sensitivity model is not claimed. At the independent center, pulmonary infection-source records (331/492) and severe pneumonia (367/492) were distinct variables. All named infection-source indicators were unrecorded in 80/492 patients; the 94/492 ‘other or unknown’ category operationally denotes no named source indicator and does not establish absence of infection.

Supplementary Table S16. Overlap between SOFA-2 cardiovascular score and five-agent norepinephrine-equivalent burden in the MIMIC-IV 24-hour landmark cohort

**(A). Locked cardiovascular scoring rules aligned with the JAMA 2025 framework**

| **Score** | **Locked SOFA-2 cardiovascular rule aligned with the JAMA 2025 framework** |
| --- | --- |
| 0 | No qualifying vasopressor/inotrope or mechanical support and MAP ≥70 mmHg; the locked SQL also retains score 0 when MAP is unavailable. |
| 1 | No qualifying vasopressor/inotrope or mechanical support and minimum MAP <70 mmHg. |
| 2 | Norepinephrine + epinephrine >0 to ≤0.2 μg/kg/min without another agent; or any qualifying other agent not meeting a higher category; dopamine-only ≤20 μg/kg/min. |
| 3 | Norepinephrine + epinephrine >0.2 to ≤0.4 μg/kg/min without another agent; or >0 to ≤0.2 μg/kg/min plus another agent; or dopamine >20 to ≤40 μg/kg/min. |
| 4 | Norepinephrine + epinephrine >0.4 μg/kg/min; or >0.2 to ≤0.4 μg/kg/min plus another agent; or dopamine >40 μg/kg/min; or qualifying mechanical circulatory support/VA or VAV-ECMO. |

**(B). Cross-tabulation of SOFA-2 cardiovascular score and five-agent NE-equivalent burden**

| **SOFA-2 cardiovascular score** | **NE-equivalent ≤0.1** | **NE-equivalent >0.1–0.3** | **NE-equivalent >0.3–0.5** | **NE-equivalent >0.5** | **Total** |
| --- | --- | --- | --- | --- | --- |
| 0 | 48 (84.2%) | 8 (14.0%) | 1 (1.8%) | 0 (0.0%) | 57 |
| 1 | 707 (77.9%) | 170 (18.7%) | 18 (2.0%) | 12 (1.3%) | 907 |
| 2 | 2,784 (43.3%) | 3,256 (50.7%) | 253 (3.9%) | 130 (2.0%) | 6,423 |
| 3 | 130 (6.2%) | 1,357 (64.4%) | 516 (24.5%) | 103 (4.9%) | 2,106 |
| 4 | 84 (3.5%) | 232 (9.7%) | 661 (27.6%) | 1,420 (59.2%) | 2,397 |

Supplementary Table S16 (continued).

**(C). Continuous vasoactive burden and observed outcomes by cardiovascular score**

| **SOFA-2 cardiovascular score** | **Outcome/drug-use N** | **NE-equivalent, median [IQR], μg/kg/min (finite burden)** | **Post-landmark mortality** | **Multi-vasopressor use** | **Vasopressin use** |
| --- | --- | --- | --- | --- | --- |
| 0 | 57 | 0.050 [0.030, 0.060] | 5/57 (8.8%) | 1.8% | 3.5% |
| 1 | 909 | 0.050 [0.030, 0.080] | 59/909 (6.5%) | 1.4% | 0.3% |
| 2 | 6,423 | 0.100 [0.060, 0.150] | 1,061/6,423 (16.5%) | 9.1% | 1.9% |
| 3 | 2,106 | 0.231 [0.200, 0.300] | 518/2,106 (24.6%) | 55.9% | 15.6% |
| 4 | 2,397 | 0.540 [0.400, 0.842] | 1,044/2,397 (43.6%) | 78.7% | 58.2% |

**(D). Quantification of overlap**

| **Overlap metric** | **Estimate** |
| --- | --- |
| Spearman correlation between cardiovascular score and continuous five-agent NE-equivalent burden | ρ=0.727; P<0.001 |
| Association between cardiovascular score and four-level NE-equivalent stratum | Cramer's V=0.492; P<0.001 |
| Cardiovascular score 3-4 with NE-equivalent ≤0.1 | 214/4,503 (4.8%) |
| NE-equivalent >0.5 with cardiovascular score 0-2 | 142/1,665 (8.5%) |

Notes. Panel A summarizes the locked SOFA-2 cardiovascular scoring rules. Qualifying infusion episodes lasted at least 1 hour. “Another agent” denotes dopamine, dobutamine, vasopressin, phenylephrine or milrinone; dopamine also has >20 and >40 μg/kg/min exceptions. Mechanical circulatory support includes VA/VAV-ECMO, IABP, LVAD and microaxial-flow pumps. The locked implementation assigns the maximum score reached across qualifying states. Panel B and the burden-based calculations in Panel D use the 11,890 landmark patients with finite burden available, including 2,686 post-landmark deaths by ICU day 28. Panel C reports mortality, multi-vasopressor use and vasopressin use in all 11,892 landmark patients (2,687 deaths), whereas its median [IQR] burden summaries use the 11,890 patients with finite burden. Both missing burden values occurred at cardiovascular score 1; therefore, Panel C uses N=909 for score-1 outcomes and n=907 for its burden summary. The five-agent peak burden was calculated as norepinephrine + epinephrine + dopamine/100 + phenylephrine/10 + vasopressin (U/min) ×2.5. Panel B cross-tabulates cardiovascular scores and the four study-defined burden strata; Panel C reports continuous burden and observed outcomes by cardiovascular score; Panel D reports Spearman correlation, Cramer's V and discordant high-support classifications. The two measures share treatment-intensity information but differ in dose and support-rule granularity. CI, confidence interval; ECMO, extracorporeal membrane oxygenation; IABP, intra-aortic balloon pump; ICU, intensive care unit; IQR, interquartile range; LVAD, left ventricular assist device; MAP, mean arterial pressure; SOFA, Sequential Organ Failure Assessment; VA/VAV-ECMO, venoarterial/venoarterial-venous extracorporeal membrane oxygenation.

Supplementary Table S17. Total-score and non-cardiovascular SOFA-2 adjustment of vasoactive-support associations in complete cases

| **Exposure** | **Crude OR (95% CI)** | **Adjusted for total SOFA-2** | **Adjusted for non-cardiovascular SOFA-2** | P value for non-cardiovascular SOFA-2-adjusted model |
| --- | --- | --- | --- | --- |
| SOFA-2 cardiovascular 3–4 vs 0–2 | 2.96 (2.71–3.23) | 1.35 (1.21–1.51) | 1.90 (1.71–2.11) | <0.001 |
| NE >0.1–0.3 vs ≤0.1 | 1.89 (1.67–2.14) | 1.56 (1.36–1.80) | 1.70 (1.48–1.96) | <0.001 |
| NE >0.3–0.5 vs ≤0.1 | 4.11 (3.55–4.77) | 2.21 (1.86–2.64) | 2.84 (2.39–3.37) | <0.001 |
| NE >0.5 vs ≤0.1 | 6.17 (5.17–7.35) | 2.89 (2.43–3.44) | 4.01 (3.39–4.74) | <0.001 |

Notes. Complete-case logistic-regression models estimated associations with post-landmark 28-day all-cause mortality among MIMIC-IV patients who were alive beyond ICU admission +24 hours. Cardiovascular-score models included 10,714 patients and 2,464 events; five-agent norepinephrine-equivalent models included 10,713 patients and 2,463 events. Five-agent peak burden was calculated as norepinephrine + epinephrine + dopamine/100 + phenylephrine/10 + vasopressin (U/min) ×2.5. All adjusted models included age, sex, Charlson comorbidity index and observed lactate. “Adjusted for total SOFA-2” additionally includes the complete SOFA-2 total score; “adjusted for non-cardiovascular SOFA-2” instead includes the sum of the five non-cardiovascular domains to avoid part–whole conditioning with the cardiovascular exposure. The ≤0.1 μg/kg/min burden category and cardiovascular scores 0–2 are the reference groups. Odds ratios and 95% confidence intervals are reported for the displayed contrasts; all reported P values are <0.001. Neither adjustment strategy removes residual confounding or supports causal inference about a drug or dose strategy. CI, confidence interval; ICU, intensive care unit; NE, norepinephrine; OR, odds ratio; SOFA, Sequential Organ Failure Assessment.

Supplementary Table S18. eICU endpoint sensitivity, cohort-selection assessment, and hospital-clustered analyses

**A. Discrimination of SOFA-1 and SOFA-2 for ICU 28-day and in-hospital mortality**

| **Endpoint** | **N/events** | **SOFA-1 AUROC (cluster 95% CI)** | **SOFA-2 AUROC (cluster 95% CI)** | **Difference (cluster 95% CI)** | **Cluster‑bootstrap P** | **Paired DeLong P** |
| --- | --- | --- | --- | --- | --- | --- |
| ICU 28-day mortality | 1528/441 | 0.698 (0.662-0.731) | 0.726 (0.691-0.754) | 0.028 (0.0004-0.054) | 0.047 | 0.013 |
| In‑hospital mortality | 1525/555 | 0.689 (0.661-0.718) | 0.716 (0.688-0.743) | 0.027 (0.001-0.052) | 0.038 | 0.010 |

**B. Fixed-rule associations with mortality**

| **Endpoint** | **Predictor** | **Unadjusted OR (95% CI)** | **P value** |
| --- | --- | --- | --- |
| ICU 28-day mortality | SOFA-1 total score, per point | 1.23 (1.18-1.27) | <0.001 |
| ICU 28-day mortality | SOFA-2 total score, per point | 1.24 (1.20-1.28) | <0.001 |
| ICU 28-day mortality | SOFA-2 >=12 vs <12 | 4.16 (3.28-5.27) | <0.001 |
| ICU 28-day mortality | SOFA-2 cardiovascular 3-4 vs 0-2 | 2.29 (1.82-2.88) | <0.001 |
| In-hospital mortality | SOFA-1 total score, per point | 1.22 (1.18-1.26) | <0.001 |
| In-hospital mortality | SOFA-2 total score, per point | 1.23 (1.19-1.27) | <0.001 |
| In-hospital mortality | SOFA-2 >=12 vs <12 | 3.93 (3.12-4.94) | <0.001 |
| In-hospital mortality | SOFA-2 cardiovascular 3-4 vs 0-2 | 2.00 (1.61-2.50) | <0.001 |

**C. SOFA-2 calculability in the original 15,920-record eICU mother cohort**

| **Characteristic** | **SOFA-2 calculable (n=5,881)** | **Missing** | **SOFA-2 noncalculable (n=10,039)** | **Missing** | **SMD** |
| --- | --- | --- | --- | --- | --- |
| Age, years | 66.0 (55.0-76.0) | 0 | 68.0 (56.0-79.0) | 0 | -0.098 |
| Female sex | 2775 (47.2%) | 1 | 4861 (48.4%) | 4 | -0.025 |
| White race | 4521 (77.5%) | 44 | 7753 (77.7%) | 66 | -0.007 |
| Original SOFA score | 7.0 (4.0-10.0) | 1179 | 4.0 (2.0-6.0) | 2978 | 0.830 |
| APACHE IVa score | 78.0 (60.0-99.0) | 1670 | 62.0 (47.0-77.0) | 3772 | 0.645 |
| Native eICU/APACHE mechanical‑ventilation flag (field: ventilation; native window, not study‑defined first-24-h variable) | 2241 (38.1%) | 0 | 1323 (13.2%) | 0 | 0.596 |
| Native eICU/APACHE continuous renal‑replacement‑therapy flag (field: CRRT; native window, not study‑defined first-24-h variable) | 635 (13.5%) | 1179 | 372 (5.3%) | 2978 | 0.285 |
| Native eICU/APACHE vasopressor flag (field: vasopressor; native window, not study‑defined first-24-h variable) | 1538 (26.2%) | 0 | 1160 (11.6%) | 0 | 0.380 |
| Full‑stay ICU length of stay from unit‑discharge offset, days | 4.6 (2.4-8.7) | 0 | 2.6 (1.8-4.4) | 0 | 0.420 |
| Full‑stay hospital length of stay from hospital‑admission/discharge offsets, days | 10.3 (6.0-17.1) | 0 | 7.5 (4.7-12.7) | 0 | 0.248 |
| ICU mortality | 1084 (18.4%) | 0 | 684 (6.8%) | 3 | 0.355 |
| In‑hospital mortality | 1480 (25.4%) | 53 | 1272 (12.8%) | 113 | 0.324 |

Supplementary Table S18 (continued).

**D. Inclusion in the strict eICU transportability cohort**

| **Characteristic** | **Strict transportability cohort (n=1,528)** | **Missing** | **Not included in strict cohort (n=14,392)** | **Missing** | **SMD** |
| --- | --- | --- | --- | --- | --- |
| Age, years | 68.0 (57.0-78.0) | 0 | 67.0 (55.0-78.0) | 0 | 0.061 |
| Female sex | 746 (48.8%) | 0 | 6890 (47.9%) | 5 | 0.019 |
| White race | 1214 (80.1%) | 13 | 11060 (77.4%) | 97 | 0.068 |
| Original SOFA score | 10.0 (7.0-12.0) | 0 | 5.0 (3.0-7.0) | 4157 | 1.506 |
| APACHE IVa score | 91.0 (72.0-113.0) | 131 | 65.0 (49.0-81.0) | 5311 | 0.965 |
| Native eICU/APACHE mechanical‑ventilation flag (field: ventilation; native window, not study‑defined first-24-h variable) | 825 (54.0%) | 0 | 2739 (19.0%) | 0 | 0.779 |
| Native eICU/APACHE continuous renal‑replacement‑therapy flag (field: CRRT; native window, not study‑defined first-24-h variable) | 292 (19.1%) | 0 | 715 (7.0%) | 4157 | 0.366 |
| Native eICU/APACHE vasopressor flag (field: vasopressor; native window, not study‑defined first-24-h variable) | 841 (55.0%) | 0 | 1857 (12.9%) | 0 | 0.993 |
| Study‑defined first-24-h vasoactive‑drug exposure | 1,528/1,528 (100.0%) | 0 | Not applicable | Not applicable | Not applicable |
| Full‑stay ICU length of stay from unit‑discharge offset, days | 4.5 (2.4-8.4) | 0 | 2.9 (1.8-5.5) | 0 | 0.246 |
| Full‑stay hospital length of stay from hospital‑admission/discharge offsets, days | 9.1 (5.1-15.2) | 0 | 8.2 (5.0-14.4) | 0 | 0.024 |
| ICU mortality | 450 (29.5%) | 0 | 1318 (9.2%) | 3 | 0.532 |
| In‑hospital mortality | 555 (36.4%) | 3 | 2197 (15.4%) | 163 | 0.492 |

Supplementary Table S18 (continued).
E. Hospital distribution in the strict eICU cohort

| **Metric** | **Value** |
| --- | --- |
| Hospitals | 99 |
| Patients per hospital, median (IQR) | 8 (2-21) |
| Hospitals with <5 patients | 37 (37.4%) |
| Largest hospital contribution | 105 (6.9%) |
| In‑hospital deaths | 555/1525 (36.4%) |

Notes: Values are median (IQR) or n (%), calculated among nonmissing observations; missing counts are shown separately. The original mother cohort comprised 15,920 eICU records; SOFA-2 was calculable under the database implementation in 5,881 records and noncalculable in 10,039. The strict transportability cohort comprised 1,528 patients from 99 hospitals after applying the structured sepsis diagnosis, organ-dysfunction, vasopressor-exposure, and complete-scoring criteria. For the derived ICU 28-day endpoint, patients discharged alive from the ICU before day 28 were classified as alive at day 28. In-hospital mortality was defined directly from hospitaldischargestatus; three patients with missing hospital discharge status were excluded from that endpoint only. AUROC difference was SOFA-2 minus SOFA-1. Cluster-bootstrap 95% CIs and P values were based on 2,000 resamples of hospitals. Odds ratios in Panel B are unadjusted fixed-rule associations; the existing hospital distribution and cohort-selection panels are retained for context. Absolute SMD values >0.10 indicate potentially meaningful imbalance. Abbreviations: APACHE, Acute Physiology and Chronic Health Evaluation; AUROC, area under the receiver operating characteristic curve; CI, confidence interval; CRRT, continuous renal replacement therapy; IQR, interquartile range; OR, odds ratio; SMD, standardized mean difference; SOFA, Sequential Organ Failure Assessment. The native eICU/APACHE flags reported in Panels C and D were used only for cohort-selection comparisons and are not identical to the study-defined first-24-h treatment variables reported in main Table 1. By design, all 1,528 patients in the strict transportability cohort met the study-defined first-24-h vasoactive-exposure criterion. The native comparison fields were ventilation, CRRT, and vasopressor in input_eicu_sepsis.csv; their source extraction did not provide a harmonized ICU-hours 0–24 window. ICU length of stay was derived from the unit-discharge offset, whereas hospital length of stay was derived from hospital admission and discharge offsets; both describe the full recorded stay rather than a first-24-h treatment window.

Supplementary Table S19. Redundancy, coefficients and internal performance of the vasoactive-drug composition model

**A. Deterministic redundancy and design-matrix rank assessment**

| **Assessment item** | **Observed result** | **Interpretation** |
| --- | --- | --- |
| Drug‑class count equals the sum of the five drug indicators | 11,997/11,997 | Exact identity |
| Multi‑vasopressor use equals I(sum of five indicators ≥2) | 11,997/11,997 | Exact identity |
| Original design matrix | 9 columns; rank 8 | Rank deficient |
| Aliased coefficient in the original fit | Vasopressin indicator | Implicitly set to NA by model fitting |
| Revised design matrix | 7 columns; rank 7 | Full rank |

**B. Revised model coefficients and variance inflation factors**

| **Predictor** | **Beta** | **SE** | **Adjusted OR (95% CI)** | **P value** | **VIF** |
| --- | --- | --- | --- | --- | --- |
| Intercept | -3.501 | 0.091 | 0.03 (0.03–0.04) | <0.001 | — |
| SOFA-2 total, per point | 0.209 | 0.008 | 1.23 (1.21–1.25) | <0.001 | 1.36 |
| Norepinephrine use | 0.729 | 0.075 | 2.07 (1.79–2.40) | <0.001 | 2.34 |
| Epinephrine use | -0.550 | 0.080 | 0.58 (0.49–0.67) | <0.001 | 1.07 |
| Dopamine use | 0.689 | 0.090 | 1.99 (1.67–2.38) | <0.001 | 1.06 |
| Phenylephrine use | -0.275 | 0.064 | 0.76 (0.67–0.86) | <0.001 | 2.09 |
| Vasopressin use | 0.313 | 0.066 | 1.37 (1.20–1.56) | <0.001 | 1.34 |

**C. Apparent and internally validated model performance**

| **Model** | **N** | **Events** | **AUROC (95% CI)** | **Brier score** |
| --- | --- | --- | --- | --- |
| SOFA-2 total | 11,890 | 2,686 | 0.731 (0.720–0.742) | 0.151 |
| SOFA-2 total + five drug indicators | 11,890 | 2,686 | 0.768 (0.758–0.778) | 0.146 |
| Bootstrap-corrected composition model | 11,890 | 2,686 | 0.767 | 0.146 |

Supplementary Table S19 (continued). D. Internal calibration of the drug-composition model

| **Analysis** | **N/events** | **Calibration intercept** | **Calibration slope** | **Brier score** | **O/E** |
| --- | --- | --- | --- | --- | --- |
| Apparent development-sample | 11,890/2,686 | 0.000 | 1.000 | 0.146 | 1.000 |
| 1,000-bootstrap corrected | 11,890/2,686 | 0.001 | 0.998 | 0.146 | — |

The composition model had an apparent AUROC of 0.768 and Brier score of 0.146; bootstrap-corrected values were 0.767 and 0.146. Apparent calibration was ideal by construction in the development data; bootstrap-corrected intercept and slope were 0.001 and 0.998. These internal estimates do not demonstrate calibration in a new cohort.

Notes. Panel A was audited in the 11,997-patient exposure-eligible source cohort; Panels B–D used the 11,890-patient common MIMIC-IV prediction subset with 2,686 post-landmark deaths. The logistic model includes SOFA-2 total and five non-mutually-exclusive first-24-hour indicators for norepinephrine, epinephrine, dopamine, phenylephrine and vasopressin. Drug-class count and multi-vasopressor use were excluded because they are deterministic functions of these five indicators. Each drug coefficient is conditional on SOFA-2 total and the other drug indicators and must not be interpreted as relative efficacy, protection, or harm. Panel D reports apparent development-sample calibration and bootstrap optimism-corrected intercept and slope; apparent ideal calibration is expected after fitting and is not external validation. This exploratory model is not a deployable clinical calculator. AUROC, area under the receiver operating characteristic curve; CI, confidence interval; ICU, intensive care unit; O/E, observed-to-expected ratio; SOFA, Sequential Organ Failure Assessment.

Supplementary Table S20. Multiple-imputation primary analyses and complete-case sensitivity analyses for post-landmark 28-day mortality

| **Analysis** | **Contrast** | **MI N/events** | **MI adjusted OR (95% CI)** | **Complete-case N/events** | **Complete-case adjusted OR (95% CI)** | **Relative OR change** | **Conclusion** |
| --- | --- | --- | --- | --- | --- | --- | --- |
| SOFA-2 threshold | SOFA-2 total ≥12 vs <12 | 11,892/2,687 | 3.89 (3.44–4.39) | 10,714/2,464 | 3.90 (3.45–4.42) | −0.4% | Direction unchanged |
| SOFA-2 cardiovascular | Score 3–4 vs 0–2; non-CV adjustment | 11,892/2,687 | 1.82 (1.64–2.01) | 10,714/2,464 | 1.90 (1.71–2.11) | −4.3% | Direction unchanged |
| NE-equivalent strata | >0.1–0.3 vs ≤0.1; non-CV adjustment | 11,890/2,686 | 1.57 (1.38–1.79) | 10,713/2,463 | 1.70 (1.48–1.96) | −7.6% | Direction unchanged |
| NE-equivalent strata | >0.3–0.5 vs ≤0.1; non-CV adjustment | 11,890/2,686 | 2.68 (2.28–3.15) | 10,713/2,463 | 2.84 (2.39–3.37) | −5.5% | Direction unchanged |
| NE-equivalent strata | >0.5 vs ≤0.1; non-CV adjustment | 11,890/2,686 | 3.60 (3.07–4.23) | 10,713/2,463 | 4.01 (3.39–4.74) | −10.2% | Direction unchanged |

Note. Patients were required to be alive beyond ICU admission +24 hours, and the outcome was death by ICU day 28. In the multiple-imputation analyses, lactate was imputed by predictive mean matching in 20 datasets; outcomes, SOFA scores and vasoactive exposures were retained as observed. The SOFA-2 ≥12 model adjusted for age, sex, Charlson comorbidity index and lactate. Cardiovascular-score and five-agent norepinephrine-equivalent models adjusted for age, sex, Charlson comorbidity index, lactate and the sum of the five non-cardiovascular SOFA-2 domains. Five-agent peak burden was calculated as norepinephrine + epinephrine + dopamine/100 + phenylephrine/10 + vasopressin (U/min) ×2.5. Primary imputed models included 11,892 patients/2,687 events for the SOFA-2 threshold and cardiovascular score and 11,890/2,686 for burden models. Complete-case models used the same outcome, contrasts and adjustment sets and included 10,714/2,464 for the threshold and cardiovascular score and 10,713/2,463 for burden models. Relative OR changes and the direction of each association are reported as shown. These sensitivity analyses address missing lactate but do not remove residual confounding or establish causal treatment effects. CI, confidence interval; ICU, intensive care unit; MI, multiple imputation; NE, norepinephrine; OR, odds ratio; SOFA, Sequential Organ Failure Assessment. Relative OR change was calculated as 100 × (multiple-imputation OR − complete-case OR) / complete-case OR.

Supplementary Table S21. Cohort-specific APACHE benchmarks and a simple clinical reference model

**(A). Performance of cohort-specific acute severity scores and SOFA totals on identical patient subsets**

| **Cohort** | **Model** | **N/events** | **AUROC (95% CI)** | **Apparent Brier after cohort‑specific score‑only fitting** |
| --- | --- | --- | --- | --- |
| eICU transportability cohort | APACHE IVa score | 1,392/417 | 0.686 (0.656-0.716) | 0.190 |
| eICU transportability cohort | SOFA-1 total | 1,392/417 | 0.696 (0.665-0.726) | 0.187 |
| eICU transportability cohort | SOFA-2 total | 1,392/417 | 0.725 (0.696-0.754) | 0.179 |
| Independent-center stress-test cohort | APACHE II | 492/298 | 0.658 (0.609-0.706) | 0.222 |
| Independent-center stress-test cohort | SOFA-1 total | 492/298 | 0.657 (0.609-0.705) | 0.221 |
| Independent-center stress-test cohort | SOFA-2 total | 492/298 | 0.663 (0.615-0.711) | 0.221 |

**(B). Paired AUROC comparisons against the cohort-specific APACHE score**

| **Cohort** | **Reference** | **Comparator** | **N/events** | **Reference AUROC** | **Comparator AUROC** | **AUROC difference** | **DeLong P** |
| --- | --- | --- | --- | --- | --- | --- | --- |
| eICU transportability cohort | APACHE IVa score | SOFA-1 total | 1,392/417 | 0.686 | 0.696 | +0.009 | 0.554 |
| eICU transportability cohort | APACHE IVa score | SOFA-2 total | 1,392/417 | 0.686 | 0.725 | +0.039 | 0.013 |
| Independent-center stress-test cohort | APACHE II | SOFA-1 total | 492/298 | 0.658 | 0.657 | -0.001 | 0.982 |
| Independent-center stress-test cohort | APACHE II | SOFA-2 total | 492/298 | 0.658 | 0.663 | +0.005 | 0.847 |

**(C). MIMIC-IV benchmark against the simple clinical reference model**

| **Model** | **N/events** | **AUROC (95% CI)** | **Brier score** | **Comparison** | **Paired DeLong P** |
| --- | --- | --- | --- | --- | --- |
| Simple clinical reference: age + lactate + NE burden | 10,713/2,463 | 0.715 (0.704–0.727) | 0.157 | Reference | — |
| SOFA-2 total | 10,713/2,463 | 0.737 (0.726–0.748) | 0.152 | vs clinical reference; ΔAUROC +0.022 | 0.002 |
| NE burden only | 10,713/2,463 | 0.705 (0.694–0.717) | 0.164 | vs clinical reference; ΔAUROC -0.010 | 0.071 |
| SOFA-2 total + five drug indicators | 10,713/2,463 | 0.777 (0.767–0.787) | 0.145 | vs clinical reference; ΔAUROC +0.062 | <0.001 |

**(D). All paired AUROC comparisons in the MIMIC-IV complete-case benchmark**

| **Reference** | **Comparator** | **N/events** | **Reference AUROC** | **Comparator AUROC** | **ΔAUROC** | **Paired DeLong P** |
| --- | --- | --- | --- | --- | --- | --- |
| Simple clinical reference | SOFA-2 total | 10,713/2,463 | 0.715 | 0.737 | +0.022 | 0.002 |
| Simple clinical reference | NE burden only | 10,713/2,463 | 0.715 | 0.705 | −0.010 | 0.071 |
| Simple clinical reference | SOFA-2 + five drug indicators | 10,713/2,463 | 0.715 | 0.777 | +0.062 | <0.001 |
| SOFA-2 total | NE burden only | 10,713/2,463 | 0.737 | 0.705 | −0.031 | <0.001 |
| SOFA-2 total | SOFA-2 + five drug indicators | 10,713/2,463 | 0.737 | 0.777 | +0.040 | <0.001 |
| NE burden only | SOFA-2 + five drug indicators | 10,713/2,463 | 0.705 | 0.777 | +0.072 | <0.001 |

Supplementary Table S21 (continued).

**(E). Complete-case coefficients of the simple clinical reference model**

| **Term** | **Beta** | **OR** | **95% CI** | **P value** | **N/events** | **Analysis** |
| --- | --- | --- | --- | --- | --- | --- |
| (Intercept) | -3.905 | 0.020 | 0.015-0.026 | <0.001 | 10713/2463 | Complete case |
| age | 0.026 | 1.026 | 1.023-1.030 | <0.001 | 10713/2463 | Complete case |
| lactate | 0.137 | 1.147 | 1.128-1.166 | <0.001 | 10713/2463 | Complete case |
| ne_equiv_full5 | 1.252 | 3.499 | 3.016-4.060 | <0.001 | 10713/2463 | Complete case |

Notes. Panel A compares cohort-specific acute-severity scores and SOFA totals on identical patient subsets. In eICU, APACHE IVa, SOFA-1 and SOFA-2 were evaluated in 1,392 patients with 417 database-derived ICU 28-day events. At the independent center, APACHE II, SOFA-1 and SOFA-2 were evaluated in 492 patients with 298 traceable 28-day all-cause deaths. APACHE IVa and APACHE II are not interchangeable and were evaluated only within their respective cohorts. Panel B reports paired DeLong comparisons against the applicable APACHE score. Brier score denotes mean squared prediction error. The Brier scores in Panel A are apparent values after cohort-specific score-only fitting and are not directly comparable with the fixed-model external Brier scores in main Table 4b.

Panels C–E use the same MIMIC-IV complete-case landmark subset of 10,713 patients and 2,463 post-landmark deaths by ICU day 28; no imputation was used. The simple clinical reference model contains age, observed lactate and continuous five-agent norepinephrine-equivalent burden. Panel C compares SOFA-2 total, burden alone and SOFA-2 total plus five first-24-hour drug indicators with this reference. Panel D reports all six paired AUROC comparisons among the four models, and Panel E provides the reference-model coefficients. The reference model was evaluated only in MIMIC-IV because harmonized five-agent exposure was unavailable in both external cohorts. APACHE, Acute Physiology and Chronic Health Evaluation; AUROC, area under the receiver operating characteristic curve; CI, confidence interval; ICU, intensive care unit; NE, norepinephrine; OR, odds ratio; SOFA, Sequential Organ Failure Assessment.

Supplementary Table S22. Sensitivity analysis requiring a continuous vasoactive-drug infusion of at least 60 minutes

**(A). Cohort restriction based on continuous-infusion duration**

| **Cohort** | **N** | **Deaths** | **Mortality** |
| --- | --- | --- | --- |
| Exposure-eligible source cohort | 11997 | 2792 | 23.3% |
| 24-hour landmark primary cohort | 11892 | 2687 | 22.6% |
| Duration-qualified landmark cohort | 10858 | 2611 | 24.0% |

**(B). Duration assessment in the exposure-eligible source cohort (N=11,997)**

| **Drug** | **Any recorded use, n (%)** | **Continuous infusion ≥60 min, n (%)** | **Brief or fragmented use, n** |
| --- | --- | --- | --- |
| Norepinephrine | 7,086 (59.1%) | 6,764 (56.4%) | 322 |
| Epinephrine | 1,468 (12.2%) | 1,374 (11.5%) | 94 |
| Dopamine | 759 (6.3%) | 659 (5.5%) | 100 |
| Phenylephrine | 5,901 (49.2%) | 4,895 (40.8%) | 1,006 |
| Vasopressin | 1,926 (16.1%) | 1,860 (15.5%) | 66 |

**(C). Assessment of the lowest of four study-defined dose categories of NE-equivalent burden (≤0.1 μg/kg/min)**

| **Metric** | **Primary landmark burden** | **Duration-qualified burden** |
| --- | --- | --- |
| Patients / deaths | 3753 / 424 | 3167 / 403 |
| Mortality | 11.3% | 12.7% |
| Median burden | <=0.1 | <=0.1 |
| Definition | All positive-rate intervals | Merged episode >=60 min |

**(D). Complete-case adjusted associations in the primary and duration-qualified landmark cohorts**

| **Exposure** | **Primary complete-case OR** | **Duration-qualified OR** | **P value** | **Primary N/events** | **Duration N/events** |
| --- | --- | --- | --- | --- | --- |
| SOFA-2 >=12 vs <12 | 3.90 (3.45-4.42) | 3.71 (3.27-4.20) | <0.001 | 10,714/2,464 | 9,762/2,401 |
| CV 3-4 vs 0-2 | 1.35 (1.21-1.51) | 1.30 (1.16-1.45) | <0.001 | 10,714/2,464 | 9,762/2,401 |
| NE >0.1-0.3 vs <=0.1 | 1.56 (1.36-1.80) | 1.49 (1.28-1.73) | <0.001 | 10,713/2,463 | 9,762/2,401 |
| NE >0.3-0.5 vs <=0.1 | 2.21 (1.86-2.64) | 2.49 (2.04-3.03) | <0.001 | 10,713/2,463 | 9,762/2,401 |
| NE >0.5 vs <=0.1 | 2.89 (2.43-3.44) | 2.23 (1.89-2.63) | <0.001 | 10,713/2,463 | 9,762/2,401 |

**Panel E. Non-cardiovascular SOFA-2-adjusted sensitivity estimates in the duration-qualified cohort**

| **Exposure** | **N** | **Deaths** | **Adjusted OR (95% CI)** | **P value** |
| --- | --- | --- | --- | --- |
| CV 3-4 vs 0-2 | 9,762 | 2,401 | 1.76 (1.58-1.96) | <0.001 |
| NE >0.1-0.3 vs <=0.1 | 9,762 | 2,401 | 1.57 (1.35-1.82) | <0.001 |
| NE >0.3-0.5 vs <=0.1 | 9,762 | 2,401 | 2.99 (2.46-3.65) | <0.001 |
| NE >0.5 vs <=0.1 | 9,762 | 2,401 | 2.95 (2.51-3.46) | <0.001 |

Supplementary Table S22 (continued).

**Panel F. Discrimination in the duration-qualified cohort**

| **Score** | **N** | **Deaths** | **AUROC (95% CI)** | **Paired DeLong P** |
| --- | --- | --- | --- | --- |
| SOFA-1 total | 10858 | 2611 | 0.723 (0.712-0.734) | — |
| SOFA-2 total | 10858 | 2611 | 0.723 (0.712-0.734) | 0.918 |

Note. Same-drug segments separated by ≤5 minutes were merged, and a drug exposure qualified when a merged episode lasted at least 60 minutes during the first 24 hours. The duration-qualified landmark cohort included 10,858 patients who were alive beyond ICU admission +24 hours, including 2,611 deaths by ICU day 28. Complete-case adjusted analyses included 9,762 patients and 2,401 events; the corresponding primary complete-case denominators were 10,714/2,464 for the SOFA-2 threshold and cardiovascular score and 10,713/2,463 for norepinephrine-equivalent burden. In Panel D, the SOFA-2 ≥12 models adjusted for age, sex, Charlson comorbidity index and lactate, whereas cardiovascular-score and burden models additionally used total SOFA-2 adjustment. Panel E instead adjusts the cardiovascular-score and burden models for the sum of the five non-cardiovascular SOFA-2 domains. Panel F compares SOFA-1 and SOFA-2 total-score AUROCs using a paired DeLong test in the duration-qualified cohort. The ≥12 rule and the four study-defined burden categories were retained without re-estimation. These are prognostic sensitivity analyses and do not identify causal drug effects. AUROC, area under the receiver operating characteristic curve; CI, confidence interval; CV, cardiovascular; ICU, intensive care unit; NE, norepinephrine; OR, odds ratio; SOFA, Sequential Organ Failure Assessment. The primary peak-burden exposure is the maximum simultaneous burden, not a time-weighted average, and does not summarize total exposure duration or escalation/de-escalation trajectory.

Supplementary Table S23. MIMIC-IV ICU type and admission context in the exposure-eligible source cohort and ≥60-minute duration-qualified source cohort

**(A). ICU type at first ICU admission**

| **Category** | **Original cohort, N=11,997** | **≥60-minute duration-qualified cohort, N=10,962** |
| --- | --- | --- |
| Cardiac Vascular Intensive Care Unit (CVICU) | 3,960 (33.0%) | 3,307 (30.2%) |
| Medical Intensive Care Unit (MICU) | 2,765 (23.0%) | 2,656 (24.2%) |
| Medical/Surgical Intensive Care Unit (MICU/SICU) | 1,666 (13.9%) | 1,594 (14.5%) |
| Trauma SICU (TSICU) | 1,172 (9.8%) | 1,108 (10.1%) |
| Surgical Intensive Care Unit (SICU) | 1,084 (9.0%) | 1,023 (9.3%) |
| Coronary Care Unit (CCU) | 1,070 (8.9%) | 1,009 (9.2%) |
| Neuro Surgical Intensive Care Unit (Neuro SICU) | 96 (0.8%) | 90 (0.8%) |
| Surgery/Vascular/Intermediate | 63 (0.5%) | 60 (0.5%) |
| Neuro Intermediate | 52 (0.4%) | 50 (0.5%) |
| Neuro Stepdown | 35 (0.3%) | 31 (0.3%) |
| PACU | 12 (0.1%) | 12 (0.1%) |
| Intensive Care Unit (ICU) | 9 (0.1%) | 9 (0.1%) |
| Medicine | 8 (0.1%) | 8 (0.1%) |
| Surgery/Trauma | 4 (0.0%) | 4 (0.0%) |
| Medicine/Cardiology Intermediate | 1 (0.0%) | 1 (0.0%) |

Supplementary Table S23 (continued).

**(B). Admission source**

| **Category** | **Original cohort, N=11,997** | **≥60-minute duration-qualified cohort, N=10,962** |
| --- | --- | --- |
| EMERGENCY ROOM | 4,092 (34.1%) | 3,873 (35.3%) |
| PHYSICIAN REFERRAL | 3,692 (30.8%) | 3,167 (28.9%) |
| TRANSFER FROM HOSPITAL | 3,345 (27.9%) | 3,109 (28.4%) |
| WALK-IN/SELF REFERRAL | 291 (2.4%) | 271 (2.5%) |
| PROCEDURE SITE | 199 (1.7%) | 174 (1.6%) |
| TRANSFER FROM SKILLED NURSING FACILITY | 178 (1.5%) | 172 (1.6%) |
| CLINIC REFERRAL | 103 (0.9%) | 101 (0.9%) |
| PACU | 55 (0.5%) | 53 (0.5%) |
| INFORMATION NOT AVAILABLE | 33 (0.3%) | 33 (0.3%) |
| AMBULATORY SURGERY TRANSFER | 7 (0.1%) | 7 (0.1%) |
| INTERNAL TRANSFER TO OR FROM PSYCH | 2 (0.0%) | 2 (0.0%) |

**(C). Recorded admission type**

| **Category** | **Original cohort, N=11,997** | **≥60-minute duration-qualified cohort, N=10,962** |
| --- | --- | --- |
| EW EMER. | 5,236 (43.6%) | 4,964 (45.3%) |
| URGENT | 2,682 (22.4%) | 2,463 (22.5%) |
| SURGICAL SAME DAY ADMISSION | 1,623 (13.5%) | 1,329 (12.1%) |
| OBSERVATION ADMIT | 1,194 (10.0%) | 1,123 (10.2%) |
| ELECTIVE | 813 (6.8%) | 682 (6.2%) |
| DIRECT EMER. | 448 (3.7%) | 400 (3.6%) |
| DIRECT OBSERVATION | 1 (0.0%) | 1 (0.0%) |

Supplementary Table S23 (continued).

**(D). Administrative elective/emergency grouping**

| **Category** | **Original cohort, N=11,997** | **≥60-minute duration-qualified cohort, N=10,962** |
| --- | --- | --- |
| Elective | 813 (6.8%) | 682 (6.2%) |
| Emergency/urgent | 8,366 (69.7%) | 7,827 (71.4%) |
| Observation/same-day admission | 2,818 (23.5%) | 2,453 (22.4%) |
| Other recorded admission type | 0 (0.0%) | 0 (0.0%) |
| Missing | 0 (0.0%) | 0 (0.0%) |

Notes. This table contains MIMIC-IV cohorts only. ICU type is reported as the exact administrative first-care-unit label. ICU type was not used to infer individual surgical-admission or postoperative status. Admission source and admission type are administrative categories. The elective/emergency grouping is derived only from exact admission_type labels using the stated rule: ELECTIVE was classified as elective; EW EMER., DIRECT EMER., and URGENT as emergency/urgent; DIRECT OBSERVATION, OBSERVATION ADMIT, and SURGICAL SAME DAY ADMISSION as observation/same-day admission; all other nonmissing labels as other recorded admission type; and missing or empty labels as missing. Direct surgical-admission status and postoperative status were not reliably available from the available analysis dataset. SURGICAL SAME DAY ADMISSION is an administrative admission-type label and does not by itself establish postoperative status. Percentages use the corresponding cohort denominator. Categories are mutually exclusive within each panel.

Supplementary Table S24. Incremental prediction models for post-landmark 28-day all-cause mortality in MIMIC-IV

| **Model** | **Key predictors** | **N/events** | **Apparent AUROC (95% CI)** | **Brier score** | **Comparator** | **ΔAUROC** | **Paired DeLong P** | **LRT P** | **Corrected AUROC** | **Corrected Brier** |
| --- | --- | --- | --- | --- | --- | --- | --- | --- | --- | --- |
| M0 | Non-CV SOFA-2 | 11,890/2,686 | 0.708 (0.696–0.719) | 0.155 | — | — | — | — | — | — |
| M1 | M0 + CV score | 11,890/2,686 | 0.739 (0.728–0.750) | 0.150 | M0 | +0.031 | <0.001 | <0.001 | — | — |
| M2 | M0 + continuous burden | 11,890/2,686 | 0.735 (0.724–0.746) | 0.152 | M0 | +0.027 | <0.001 | <0.001 | — | — |
| M3 | M0 + CV score + burden | 11,890/2,686 | 0.743 (0.732–0.754) | 0.150 | M0 | +0.035 | <0.001 | <0.001 | — | — |
| M4 | M0 + five drug indicators | 11,890/2,686 | 0.763 (0.753–0.773) | 0.147 | M0 | +0.055 | <0.001 | <0.001 | — | — |
| M5 | Complete SOFA-2 total | 11,890/2,686 | 0.731 (0.720–0.742) | 0.151 | M0 | +0.024 | <0.001 | — | 0.731 | 0.151 |
| Continuous five-agent NE burden only | landmark_event ~ ne_equiv_full5 | 11,890/2,686 | 0.694 (0.683–0.706) | 0.164 | M5 (SOFA-2 total alone) | −0.037 | <0.001 | — | — | — |
| SOFA-2 total + continuous five-agent NE burden | landmark_event ~ sofa2_total + ne_equiv_full5 | 11,890/2,686 | 0.742 (0.731–0.753) | 0.150 | M5 (SOFA-2 total alone) | +0.011 | <0.001 | <0.001 | — | — |
| composition | SOFA-2 total + five drug indicators | 11,890/2,686 | 0.768 (0.758–0.778) | 0.146 | M5 | +0.037 | <0.001 | <0.001 | 0.767 | 0.146 |

Note. The common complete prediction subset included 11,890 patients who were alive beyond ICU admission +24 hours and had complete prediction variables, including 2,686 deaths by ICU day 28. M0 contains the five non-cardiovascular SOFA-2 domains; M1 adds the cardiovascular score; M2 adds continuous five-agent norepinephrine-equivalent burden; M3 adds both cardiovascular score and continuous burden; M4 adds five non-mutually-exclusive first-24-hour indicators for norepinephrine, epinephrine, dopamine, phenylephrine and vasopressin; M5 contains complete SOFA-2 total; and the composition model contains SOFA-2 total plus the five drug indicators. The two Figure 5/Supplementary Figure S5 models added here were fitted as landmark_event ~ ne_equiv_full5 and landmark_event ~ sofa2_total + ne_equiv_full5, respectively, by logistic regression on the same complete prediction subset. Their displayed predictions, without refitting or reparameterization, were used in decision-curve analysis together with M5 and the composition model. ΔAUROC and paired DeLong P values compare each model with the stated comparator. M3 had an AUROC of 0.743 versus 0.739 for M1; the descriptive difference was 0.004, and no direct M3-versus-M1 paired test was prespecified. Likelihood-ratio tests are reported only for nested comparisons. M5 and the composition model underwent 1,000-resample bootstrap optimism correction. These internally evaluated prediction models support comparative prognostic assessment and do not define a treatment strategy or a deployable calculator. AUROC, area under the receiver operating characteristic curve; CV, cardiovascular; DCA, decision-curve analysis; ICU, intensive care unit; LRT, likelihood-ratio test; NE, norepinephrine; SOFA, Sequential Organ Failure Assessment.
